# Temporal Dynamics Between State Attachment Security, Avoidance, and Anxiety: Insights into Everyday Attachment System Functioning

**DOI:** 10.1177/01461672251333472

**Published:** 2025-05-21

**Authors:** Jaakko Tammilehto, Aleksandra Kaurin, Peter Kuppens, Guy Bosmans, Mervi Vänskä, Marjo Flykt, Kirsi Peltonen, Jallu Lindblom

**Affiliations:** 1 Tampere University, Finland; 2 University of Helsinki, Finland; 3 University of Wuppertal, North Rhine-Westphalia, Germany; 4 KU Leuven, Belgium; 5 University of Turku, Finland

**Keywords:** attachment, state attachment, ecological momentary assessment, experience sampling method, dynamic structural equation model

## Abstract

The attachment system regulates behavior to maintain security and cope with insecurities. Although this necessitates the coordination of different attachment states, research on state-level dynamics is scarce. We used data from two ecological momentary assessment studies (*N*s = 122 and 127) to examine cross-lagged effects between state attachment security, avoidance, and anxiety. We hypothesized dampening effects between the secure and insecure states. Furthermore, we expected trait-level attachment to moderate the state-level dynamics. Attachment states were assessed seven or ten times daily over a week. Trait attachment was assessed using the Experiences in Close Relationships–Revised. Results showed that state security predicted decreased state avoidance and anxiety. Evidence also emerged for state avoidance predicting decreased security. Trait attachment showed no expected moderation effects on the state-level dynamics. Our study underscores the predominance of security over insecurities, suggesting that the functioning of the everyday attachment system centers on fluctuations in the sense of security.

## Introduction

The attachment system drives individuals to seek proximity and protection from their attachment figures (Bowlby, 1982). Research has demonstrated that the system is activated by cues of threats in both children and adults (Ainsworth et al., 1978; Mikulincer & Shaver, 2023). Research also suggests that individuals’ trait attachment, consisting of relatively stable beliefs and expectations about their own safety and others’ availability, shapes the dynamics of the attachment system (Mikulincer & Shaver, 2023). However, only recent research has begun to explore the real-time fluctuations of attachment states—security, avoidance, and anxiety—in daily life (Kaurin et al., 2022; Tammilehto et al., 2022, Tammilehto, Kaurin, et al., 2025). State attachment refers to momentary experiences of (in)security, stemming from the activation of specific parts of attachment representations (Bosmans et al., 2020; Gillath et al., 2009). Despite the recent efforts, it remains unclear whether and how different attachment states coordinate with each other. Knowledge about their temporal interplay would help better understand the dynamic organization of the attachment system, shedding light on how attachment responses fluctuate and evolve over time. In this ecological momentary assessment (EMA) study, we investigate the cross-lagged effects between different attachment states to gain a deeper understanding of the temporal dynamics within everyday attachment system functioning.

### Relatively Stable Trait Attachment

Attachment research has traditionally examined adult attachment from a trait perspective, focusing on the relatively stable, prototypical attachment representations that guide individuals’ behaviors (Fraley et al., 2011; Mikulincer & Shaver, 2023). Empirical research has identified individual differences in trait attachment along two dimensions of avoidance and anxiety, with low levels of both reflecting trait attachment security (Fraley et al., 2000; Raby et al., 2021). Both dimensions reflect fundamental challenges in trusting the availability of others in times of need, yet they capture distinct forms of insecurity, each associated with unique coping strategies (Mikulincer & Shaver, 2023; Tammilehto et al., 2022, 2023).

Trait attachment avoidance is marked by persistent expectations of others’ unavailability, coupled with a reluctance to seek and receive support (Fraley et al., 2000). Consequently, avoidantly attached individuals employ deactivating strategies that inhibit the activation of the attachment system, such as defensive self-reliance and minimization of emotions (Mikulincer & Shaver, 2023; Tammilehto et al., 2023). In turn, trait attachment anxiety is marked by strong uncertainty about others’ availability and one’s own ability to cope with threats (Fraley et al., 2000). Consequently, anxiously attached individuals employ hyperactivating strategies that accelerate the activation of the attachment system, such as intensifying negative emotions and ruminating and catastrophizing threats (Mikulincer & Shaver, 2023; Tammilehto et al., 2022, 2023). Finally, low levels of both trait attachment avoidance and anxiety indicate trait security, characterized by positive expectations about others’ availability and confidence in one’s own competence (Fraley et al., 2000). Secure individuals tend to rely on secure-base strategies grounded in trust in themselves and others (Arriaga et al., 2018; Mikulincer & Shaver, 2023). Meta-analytical evidence links high levels of both trait dimensions to various mental health, relationship, and emotion regulation challenges (Candel & Turliuc, 2019; X. Zhang et al., 2022). Notably, each dimension remains associated with these challenges even after accounting for the other (X. Zhang et al., 2022).

### Dynamic State Attachment

While trait attachment reflects individuals’ predominant beliefs and expectations, individuals also possess more nuanced attachment representations (Arriaga et al., 2018; Baldwin et al., 1996; Bosmans et al., 2020; Fraley, 2007). These representations, formed over the course of intimate relationship histories, encompass both secure and insecure elements (Arriaga et al., 2018; Bosmans et al., 2020). Consequently, everyone can occasionally experience shifts between feelings of security and insecurity, regardless of their trait attachment (Arriaga et al., 2018; Mikulincer & Shaver, 2016). These moment-to-moment fluctuations in state attachment reflect adaptive responses of the attachment system, arising from the interplay between representations and situational cues (Bosmans et al., 2020; Gillath et al., 2009).

Deviating from the two-dimensional model of trait attachment (Fraley et al., 2000; Raby et al., 2021), research has identified three state attachment dimensions that more accurately describe the dynamics of fluctuating attachment experiences (Bosmans et al., 2014; Gillath et al., 2009; Kaurin et al., 2022; Stöven & Herzberg, 2021; Trentini et al., 2015). State security reflects the sense of being loved and cared for, which is the primary set goal of the attachment system (Gillath et al., 2009; Tammilehto et al., 2022). In contrast, state avoidance embodies the fear of losing independence and the deactivation of the attachment system to avoid distress related to the perceived unavailability of the attachment figure. Finally, state anxiety reflects an intense need to feel loved and cared for and the hyperactivation of the attachment system to enhance proximity to the attachment figure (Gillath et al., 2009; Tammilehto et al., 2022).

Although research on state attachment is relatively new, evidence of its momentary changes is available from studies utilizing experimental, diary, and EMA designs. First, experimental studies with children (Cuyvers et al., 2023; Vandevivere et al., 2018) and adults (Bosmans et al., 2014; Gillath et al., 2009) indicate that positive thoughts about others’ availability may foster individuals’ state security, whereas even subtle cues related to others’ unavailability can increase insecure states. Second, diary studies that measure state attachment once a day over several days suggest that state attachment fluctuates in response to a range of social experiences, such as interpersonal loss, conflict, and support during distress (Bosmans et al., 2014; Davila & Sargent, 2003; Haak et al., 2017; Verhees et al., 2023; F. Zhang, 2009). Finally, two recent adult EMA studies measuring state attachment multiple times a day over several days suggest that individuals show substantial moment-to-moment fluctuations in the state attachment dimensions that may shape and be shaped by their emotion regulation and interpersonal experiences and behaviors (Kaurin et al., 2022; Tammilehto et al., 2022).

### Temporal Dynamics Between Attachment States: A Window into System Functioning

Despite advancements in state attachment research, a gap remains in our understanding of how different state attachment dimensions influence each other over time. Uncovering such temporal cross-lagged dynamics is a central question in attachment research, with significant repercussions on various theoretical aspects of attachment theory (Arriaga et al., 2018; Bosmans et al., 2020; Long et al., 2020; Mikulincer & Shaver, 2016, 2023). First, examining state attachment cross-lags clarifies whether and how different state attachment dimensions coordinate with each other, revealing information about the normative functioning of the attachment system (Long et al., 2020; Mikulincer & Shaver, 2016, 2023). Second, comparing cross-lags between two state attachment dimensions can uncover the relative dominance of attachment states in guiding the system functioning, such as whether state security has a stronger effect on insecure states or vice versa. Finally, exploring the associations of trait attachment with state attachment cross-lags enables researchers to consider how individuals’ prototypical attachment representations might translate into the dynamics of the attachment system (Arriaga et al., 2018; Bosmans et al., 2020; Mikulincer & Shaver, 2023).

Although no previous studies exist on state attachment cross-lags, hypotheses can be derived from the prevailing models of the attachment system. Given that the desired set goal of the attachment system is to attain a sense of security, high state attachment security can generally decrease subsequent avoidance and anxiety (Long et al., 2020; Mikulincer & Shaver, 2016, 2023). Low security, in turn, can lead to an increase in avoidance and anxiety, reflecting the activation of the attachment system’s secondary strategies when the primary secure-base strategies are not viable (Long et al., 2020; Mikulincer & Shaver, 2016, 2023). Conversely, as state avoidance and anxiety indicate distrust or uncertainty in attaining security, high state avoidance, and anxiety may generally reduce individuals’ security through various explicit and implicit socioemotional processes (Long et al., 2020; Mikulincer & Shaver, 2016, 2023).

The question of the relative dominance of the cross-lagged effects—whether one state attachment dimension has a stronger effect on another or vice versa—is more complex. On the one hand, high security might have larger effects on decreased avoidance and anxiety than the reverse due to the motivational drive of the attachment system to resist change when feeling secure (Long et al., 2020; Mikulincer & Shaver, 2016, 2023). On the other hand, humans have evolved to exhibit heightened sensitivity to prioritize negative information over positive (Nesse, 2005; Norris, 2021; Vaish et al., 2008). This might result in larger effects of avoidance and anxiety on security than the reverse (Bosmans et al., 2020).

Finally, differences in trait attachment dimensions may be crucial in moderating the cross-lags related to the corresponding state dimension. Individuals with high trait attachment avoidance and/or anxiety may show strong resistance to security after experiencing high levels of insecure state dimensions (i.e., avoidance or anxiety) that align with their trait-like beliefs and expectations (Arriaga et al., 2018; Bosmans et al., 2020). They may also struggle to maintain security (Arriaga et al., 2018; Bosmans et al., 2020). Thus, high trait avoidance may strengthen the effects of state avoidance on decreased security while weakening the effect of security on decreased avoidance. Following the same rationale, trait attachment anxiety may play a comparable role in the cross-lags between state anxiety and security. As such, beyond testing the average cross-lagged effects, it is crucial to take initial steps toward understanding how trait attachment might moderate these state-level dynamics.

### Current Study

**Figure 1.**
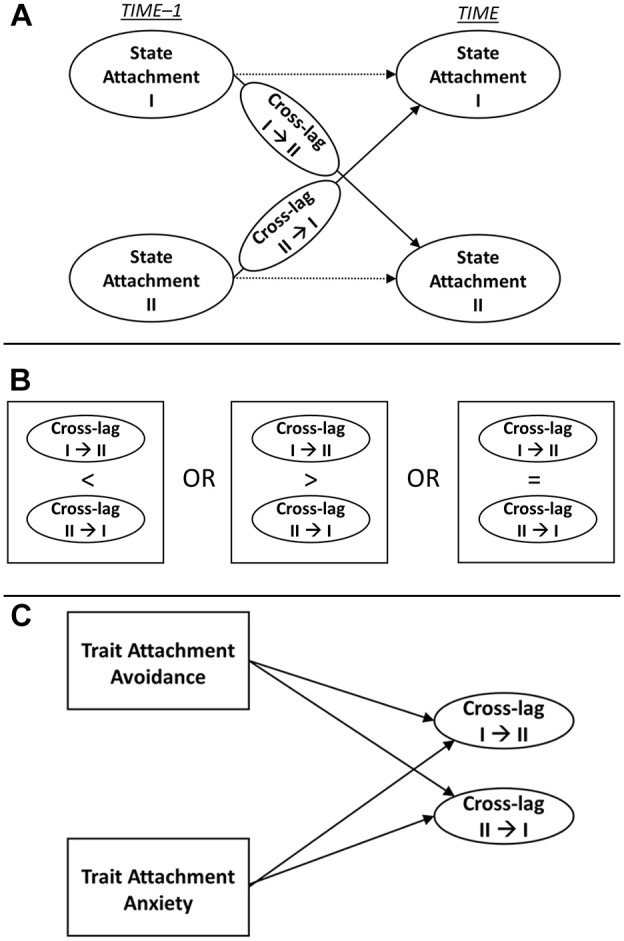
Conceptual overview of study: (A) state attachment cross-lags, (B) hypothetical possibilities for their relative dominance, and (C) role of trait attachment in cross-lags. *Note.* The Greek numbers I and II refer to separate state attachment dimensions (e.g., security and avoidance).

In this EMA study, with a preregistered analysis plan and hypotheses, we aimed to examine the cross-lagged effects among state attachment dimensions. To enhance the robustness and rigor of our study design, we used data from two independent adult samples and applied dynamic structural equation models (DSEM; Asparouhov et al., 2018), allowing us to simultaneously analyze multivariate state-level dynamics. Figure 1 depicts a conceptual overview of our study, which addressed three separate but interrelated research questions.

First, we tested the cross-lagged effects between (a) state attachment security and avoidance, (b) state attachment security and anxiety, and (c) state attachment anxiety and avoidance (Figure 1A). According to our preregistered hypotheses, high state security would predict decreased subsequent state avoidance and anxiety, while state avoidance and anxiety would predict decreased subsequent state security.

Second, we explored the relative dominance of the state attachment cross-lags (Figure 1B). Due to a lack of research and prominent theoretical predictions, we did not formulate specific hypotheses for this question. Instead, we aimed to identify consistent patterns that could inform the development of new hypotheses for future research.

Finally, we also considered the moderative role of trait attachment avoidance and anxiety in the cross-lags (Figure 1C). According to our preregistered hypotheses, high trait attachment avoidance would amplify the effect of state avoidance on decreased security and weaken the effect of security on decreased avoidance. Similarly, high trait attachment anxiety would amplify the effect of state anxiety on decreased security and weaken the effect of security on decreased anxiety.

## Methods

### Procedure and Participants

We used two independent EMA samples^1^: Sample I from the Daily Emotions research project, collected in 2017, and Sample II from the Miracles of Development research project (https://projects.tuni.fi/kehi/), collected in 2021. Using these two EMA samples allowed us to capture real-time, moment-to-moment state attachment dynamics in daily life, thereby making a unique contribution to the available empirical literature. The hypotheses and analysis plan for the study were preregistered before conducting the analyses.^2^ We conducted all preregistered analyses as planned and report them here without deviations.^3^ Additionally, we performed nonpreregistered sensitivity analyses to evaluate the robustness of our findings. These analyses are clearly labeled as “nonpreregistered” in the following sections. We report all manipulations, measures, and exclusions in this study. A comprehensive description of both Sample I and Sample II and their demographics is available in Tammilehto et al. (2023).

Sample I initially comprised 125 participants who were recruited via Tampere University email lists and paper flyers distributed on campus. The inclusion criteria were (a) being over 18 years old, (b) having the ability to use a smartphone, and (c) being fluent in Finnish. The data collection consisted of two phases: an online questionnaire phase, followed by an EMA phase about two weeks later. In the EMA phase, participants received questionnaires on their smartphones seven times a day for one week. The questionnaires were randomly assigned within seven blocks between 10:00 AM and 10:00 PM. One participant’s data from the questionnaire phase was missing, and a technical error assigned the same EMA identification number to two participants. Thus, the final sample consisted of 122 participants (*M*_age_ = 26.43 years, range: 19–52; 88.5% female). Of the participants, 53.3% were university students, 40.2% open university students, 4.1% other students, and 2.5% nonstudents. With respect to relationship status, 67.2% reported being in a romantic relationship. The successful EMA observations totaled 4,629 (77.4% compliance rate).

Sample II was an EMA subsample of Miracles of Development research project that has followed families and their children from pregnancy to early adulthood. The inclusion criteria for the EMA subsample of young adults were (a) no severe developmental disorders, (b) the availability of address information, and (c) no expressed desire to discontinue participation. Of all 710 young adults approached via mailed letters, 130 expressed their willingness to participate in the study and were thus included in this subsample. The data collection consisted of an online questionnaire, followed by an EMA phase a few days later. In the EMA phase, participants received short questionnaires on their smartphones ten times a day for 1 week. The questionnaires were randomly assigned within ten blocks between 8:00 AM and 10:00 PM. Two recruited participants provided fewer than three EMA responses (<3%), and one did not participate at all. Thus, the final sample consisted of 127 participants (*M*_age_ = 20.98, range: 20–22; 66.9% female). Of the participants, 2.4% had the highest education level of the undergraduate degree, 84.3% matriculation examination, 9.4% vocational education and training, and 3.9% comprehensive school. With respect to relationship status, 50.4% reported being in a romantic relationship. The successful EMA observations totaled 5,322 (59.9% compliance rate).

### Measures

#### State Attachment

In the EMA phases, state attachment was measured using items from the *State Adult Attachment Measure* (SAAM; Gillath et al., 2009). The SAAM is the only standard measure for adult state attachment, consisting of 21 items with a 7-point Likert scale (1 = *strongly disagree* to 7 = *strongly agree*) to assess state attachment at the current moment. The construct and predictive validity of the measure have been supported in multiple studies (Bosmans et al., 2014; Gillath et al., 2009; Kaurin et al., 2022; Stöven & Herzberg, 2021; Trentini et al., 2015).

For Sample I, we selected six items to assess state security (“I feel loved”; “I feel like I have someone to rely on”), avoidance (“If someone tried to get close to me, I would try to keep my distance”; “The idea of being emotionally close to someone makes me nervous”), and anxiety (“I feel a strong need to be unconditionally loved right now”; “I want to share my feelings with someone”) based on the factor loadings of the original validation study and the absence of strong content overlap (Gillath et al., 2009). For Sample II, the same items were used with one exception: for anxiety, “I want to talk with someone who cares for me about things that are worrying me” was used instead of “I want to share my feelings with someone.”

Multilevel confirmatory factor analyses with random intercepts indicated that the model with the three state dimensions (i.e., security, avoidance, and anxiety) at within- and between-person levels showed adequate fit: Sample I: CFI_robust_ = .949, RMSEA_robust_ = .057, SRMR_within/between_ = .056/.109; Sample II: CFI_robust_ = .990, RMSEA_robust_ = .020, SRMR_within/between_ = .016/.069. These findings are consistent with prior research, providing further support for the three-dimensional structure of state attachment (Gillath et al., 2009; Stöven & Herzberg, 2021; Trentini et al., 2015). Notably, support for the same three-dimensional structure was observed at both the within- and between-person levels, suggesting that the between-person structure of state attachment differs from the two-dimensional trait attachment structure assessed by the ECR-R (Fraley et al., 2000; Raby et al., 2021). However, evidence for the cross-level metric invariance was not generally achieved, indicating that state attachment dimensions at the between-person level may not be fully reduced back to individual attachment states (Jak & Jorgensen, 2017; Stapleton et al., 2016). A more detailed description of the measurement model results is provided in Tammilehto, Kaurin, et al. (2025).^4^

In Sample I, omega coefficients for state attachment security, avoidance, and anxiety at the within-person level were .71, .72, and .47, respectively. At the between-person level, they were .92, .97, and .70, respectively. In Sample II, same coefficients for state attachment security, avoidance, and anxiety at the within-person level were .63, .68, and .41, respectively. At the between-person level, they were .97, .98, and .83, respectively. Thus, although anxiety showed lower within-person reliabilities, all reliabilities met the benchmarks established in the EMA literature (Nezlek, 2017).

#### Trait Attachment

Trait attachment was measured using the *Experiences in Close Relationships–Revised* (ECR-R; Fraley et al., 2000). Participants reported their trait attachment avoidance (18 items; e.g., “I am nervous when partners get too close to me”) and anxiety (18 items; e.g., “I get uncomfortable when a romantic partner wants to be very close”) using a 7-point Likert scale (1 = *strongly disagree* to 7 = *strongly agree*). In Sample I and Sample II, Cronbach’s alphas were .91 and .91 for trait attachment avoidance and .92 and .93 for trait attachment anxiety, respectively.

#### Covariates

For associations of trait attachment, we controlled for the proportion of participants’ EMAs in which they reported being alone as well as their romantic relationship status (0 = *single*, 1 = *in a romantic relationship*). The former allowed us to exclude the possibility that general social activity would solely explain the associations of trait attachment with state attachment cross-lags, while the latter took into account the potential confounding effects of romantic relationship status on trait and state attachment. In Sample I, the time spent alone was measured in each EMA questionnaire using one item asking whether the participant was alone at the present moment (“Who are you with right now?”; 0 = *with someone*, 1 = *alone*). In Sample II, the time spent alone was measured using one item asking whether the participant had interacted with others since the previous EMA or during the last one and a half hours when the questionnaire was the first of the day (“Have you interacted with others?”; 0 = *yes, in live or virtually*, 1 = *no*).

### Statistical Analyses

We analyzed our data using DSEM (Asparouhov et al., 2018) in Mplus 8.9–8.10 (Muthén & Muthén, 2017). DSEM integrates multivariate time-series techniques with multilevel structural equation modeling, allowing individual differences in parameters (i.e., random effects). Its ability to directly model multivariate dynamics with multiple random effects makes it particularly suited for EMA research, where data consist of numerous repeated measures from multiple individuals. This approach offers distinct advantages over other common dynamic models, such as random-intercept cross-lagged panel models, which are primarily designed for panel data (Hamaker et al., 2015), and network time-series analyses, which rely on a univariate framework (Epskamp et al., 2018). Compared to traditional multilevel models, DSEM also provides robustness against various biases (e.g., Nickel’s and Lüdtke’s biases), which arise from observed mean centering (Asparouhov et al., 2018). By using latent centering, DSEM effectively decomposes the total variance of variables into within- and between-person components (Asparouhov et al., 2018).

**Figure 2.**
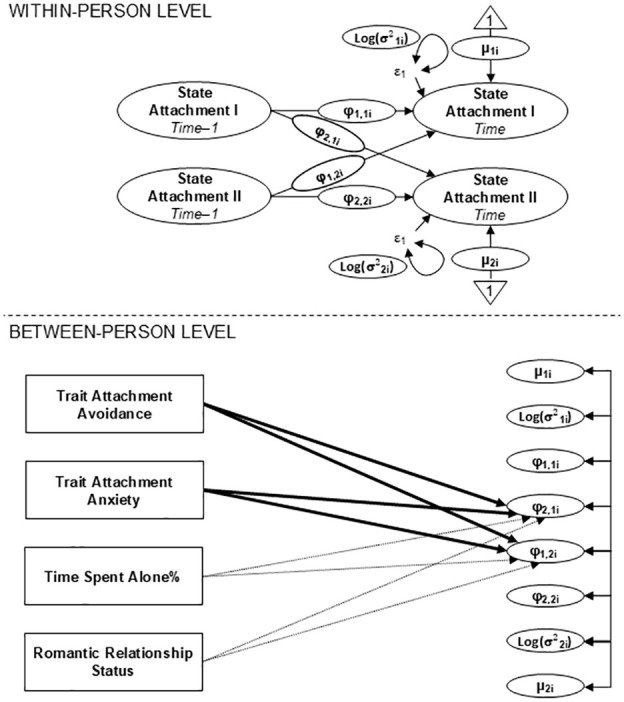
Examining state attachment cross-lags with dynamic structural equation model. *Note.* This modeling strategy was applied separately to estimate cross-lags between (a) state attachment security and avoidance, (b) state attachment security and anxiety, and (c) state attachment anxiety and avoidance. The Greek numbers I and II refer to separate state attachment dimensions (e.g., security and avoidance). Innovations were estimated using the log transformation to guarantee all individual variances to be positive (Asparouhov et al., 2018). Notably, when modeling random innovation variances, estimating only fixed covariances is not feasible, and attempting to model the random effect for this covariance often leads to convergence problems. Therefore, we preregistered a plan not to model the random covariance between the innovations. This decision simplified model components that are least relevant to our research aims and hypotheses, ensuring effective estimation. The rounded dotted arrows at the between-person level represent the modeled effects of covariates (i.e., time spent alone and romantic relationship status). Random cross-lags = φ_2,1i_ and φ_1,2i_, random intercepts/means = μ_1i_ and μ_2i_ random innovations = Log(σ²_1i_) and Log(σ²_2i_), and random autoregressive effects = φ_1,1i_, φ_2,2i_.

We modeled state attachment cross-lags in three different DSEMs. Figure 2 overviews this strategy. Related equations are presented in Supplemental Material 1. In each DSEM, we estimated the fixed and random cross-lagged effect of one state attachment dimension at the previous moment on the other state attachment dimension at the current moment and vice versa. Estimating the within-person fixed cross-lagged effects allowed us to test whether one state dimension, on average, predicts subsequent changes in another state dimension, and vice versa. This enabled us to address the first research aim, examining the cross-lagged associations between state attachment dimensions. In turn, estimating the random effects allowed each participant to get their own value for cross-lagged effects. Similarly, we estimated fixed and random intercept/mean, fixed and random innovation, and fixed and random first-order autoregressive effect of each state dimension. In principle, the optimal strategy would have been to include all state attachment dimensions into the same model. Yet, we preregistered the approach to examine cross-lagged effects in three pairs of state attachment dimensions. This decision was guided by prior research and recommendations regarding the unfeasibility of estimating numerous fixed and random effects within the same multivariate model in light of our study design and sample sizes (Asparouhov & Muthen, 2022; Schultzberg & Muthén, 2018). Our data aligned with this approach, as models incorporating all state attachment dimensions generally failed to converge. However, convergence was achieved for Sample II. Thus, we reported these results for Sample II as additional, nonpreregistered sensitivity analyses (see Results).

To assess the dominance of the cross-lags, we used the Bayesian Wald test to test whether the cross-lags could be fixed to the same value (Asparouhov & Muthén, 2021). In a more descriptive sense, we also compared the cross-lags by examining whether the 95% credible intervals (CrIs) of one standardized cross-lagged effect (e.g., the effect of security on avoidance) overlapped with the point estimate of the other standardized cross-lagged effect (e.g., the effect of avoidance on security; Schuurman et al., 2016). These procedures allowed us to assess whether there were differences in the sizes of the cross-lagged effects between state attachment dimensions.^5^ Thus, they enabled us to address the second research aim, focusing on the relative dominance of the state attachment cross-lags.

Finally, in each model, all random effects for the state attachment dimensions at the between-person level were specified to correlate. Trait attachment avoidance, anxiety, time spent alone, and romantic relationship status were specified to predict the cross-lags between the state attachment dimensions. This allowed us to explore the moderation effects of trait attachment on the cross-lags, thereby providing insights into our third research aim concerning the role of trait attachment in state attachment cross-lags.

All between-person variables were grand-mean centered, and state attachment dimensions were latent-mean centered. Bayesian Markov chain Monte Carlo estimation was used with the uninformative priors of Mplus. Two unthinned chains with 100,000 or 200,000 iterations were used in the estimation. The TINTERVAL command of Mplus was used to specify a 1-hr interval for lag interpretation. An effect was considered detected if its 95% CrIs excluded zero. In interpreting the cross-lagged effects, we used recently proposed empirical-based benchmarks for standardized cross-lagged effects: 0.03 for a small effect, 0.07 for a medium effect, and 0.12 for a large effect (Orth et al., 2024). The power simulations for the smallest detectable moderation effects of trait attachment are presented at the end of the Results section.

We first conducted the primary analyses for each individual sample, accounting for differences in study designs. Subsequently, we performed pooled nonpreregistered sensitivity analyses by combining our two samples to assess the robustness of our findings.

As preregistered sensitivity analyses, we reran all our DSEMs by specifying the alternative time interval in line with the intervals of each EMA block in the samples. In these models, state attachment at the previous assessment time was handled as the lagged observation of state attachment at the current assessment without considering differences in time lags. Lastly, we executed three preregistered DSEMs where all three state attachment dimensions at a previous moment were used to predict a single-state attachment dimension at the current moment. These analyses assessed whether the cross-lags were robust for controlling for the effect of that state attachment dimension not included in the main models.^6^

## Results

Table 1 displays the descriptive statistics and the correlations between study variables at the within-person and between-person levels. Table 2 presents the unstandardized and standardized results for Sample I concerning the cross-lagged effects between state attachment dimensions and associations of trait attachment with the cross-lags. Table 3 shows the corresponding results for Sample II.

**Table 1.** Descriptive Statistics and Correlations of Study Variables.

Descriptive statistics: Sample I							
	*n*	*M*	*SD*	Skewness	Kurtosis		ICC
1. State attachment security	4,629	5.687	1.220	−.672	−.309		.706
2. State attachment avoidance	4,629	2.376	1.331	.894	.268		.555
3. State attachment anxiety	4,629	4.200	1.318	−.025	−.371		.484
4. Trait attachment avoidance	122	2.770	.906	.345	−.683		
5. Trait attachment anxiety	122	3.255	1.187	.069	−1.074		
6. Time spent alone %	122	.375	.176	.571	−.460		
7. Romantic relationship status	122	.672	.471	−.724	−1.487		
Descriptive statistics: Sample II							
	*n*	*M*	*SD*	Skewness	Kurtosis		ICC
1. State attachment security	5,322	5.955	1.303	−1.481	2.066		.758
2. State attachment avoidance	5,322	2.390	1.410	.840	−.105		.673
3. State attachment anxiety	5,322	3.235	1.535	.421	−.333		.697
4. Trait attachment avoidance	127	3.000	1.042	.236	−.579		
5. Trait attachment anxiety	127	3.185	1.231	.503	−.527		
6. Time spent alone %	127	.125	.134	1.432	1.394		
7. Romantic relationship status	127	.504	.502	−.016	−2.015		
Correlations: Sample I (below the diagonal) and Sample II (above the diagonal)							
Within-person level	1	2	3	4			
1. State attachment security	–	−**.237**	−**.104**	.000			
2. State attachment avoidance	−**.319**	–	**.054**	−.025			
3. State attachment anxiety	**.082**	−**.227**	–	−.020			
4. Time	−**.045**	.011	−**.035**	–			
Between-person level	1	2	3	4	5	6	7
1. State attachment security	–	−**.323**	−.089	−**.411**	−**.450**	−**.387**	**.391**
2. State attachment avoidance	−**.538**	–	.073	**.471**	**.234**	.117	−**.296**
3. State attachment anxiety	.025	−.090	–	−**.201**	**.327**	−**.230**	−.045
4. Trait attachment avoidance	−**.466**	**.441**	−.138	–	**.276**	**.303**	−**.582**
5. Trait attachment anxiety	−**.443**	**.333**	**.248**	**.444**	–	.108	−**.427**
6. Time spent alone %	−**.202**	.160	−.170	.136	**.252**	–	−.161
7. Romantic relationship status	**.370**	−**.212**	−.033	−**.273**	−**.321**	−**.552**	–

*Note.* Within-person correlations are for group-mean-centered data, whereas correlations at the between-person level are for aggregated data. For bolded values, *p* < .050. *ICC* = Intraclass correlation.

**Table 2.** Sample I: Unstandardized and Standardized Cross-Lags Between State Attachment Dimensions and Associations of Trait Attachment with Cross-Lags.

Model 1: cross-lags between state attachment security and avoidance				
Within-person effects	β_unstandardized_ [95% CrI]		β_standardized_ [95% CrI]	
State attachment security → security	**0.378 [0.305, 0.446]**		**0.372 [0.328, 0.413]**	
State attachment security → avoidance	**−0.179 [−0.266, −0.094]** ^a^		**−0.117 [−0.155, −0.069]**	
State attachment avoidance → avoidance	**0.300 [0.223, 0.372]**		**0.298 [0.250, 0.343]**	
State attachment avoidance → security	−0.049 [−0.103, 0.007]		**−0.084 [−0.124, −0.050]**	
*R*^2^ state attachment security	.320			
*R*^2^ state attachment avoidance	.251			
	State attachment security → avoidance		State attachment avoidance → security	
Between-person predictors	β_unstandardized_ [95% CrI]	β_standardized_ [95% CrI]	β_unstandardized_ [95% CrI]	β_standardized_ [95% CrI]
Trait attachment avoidance	−0.071 [−0.155, 0.012]	−0.156 [−0.334, 0.026]	−0.029 [−0.091, 0.031]	−0.077 [−0.231, 0.084]
Trait attachment anxiety	−0.013 [−0.071, 0.048]	−0.037 [−0.208, 0.132]	−0.017 [−0.062, 0.026]	−0.061 [−0.208, 0.094]
% Time spent alone	0.175 [−0.263, 0.595]	0.075 [−0.108, 0.259]	0.035 [−0.271, 0.343]	0.018 [−0.137, 0.179]
Romantic relationship status	−0.070 [−0.221, 0.076]	−0.080 [−0.254, 0.084]	−0.019 [−0.138, 0.099]	−0.027 [−0.188, 0.136]
*R* ^2^	.063		.031	
Model 2: cross-lags between state attachment security and anxiety				
Within-person effects	β_unstandardized_ [95% CrI]		β_standardized_ [95% CrI]	
State attachment security → security	**0.418 [0.352, 0.480]**		**0.417 [0.380, 0.453]**	
State attachment security → anxiety	−0.047 [−0.162, 0.088]		−0.006 [−0.040, 0.029]	
State attachment anxiety → anxiety	**0.326 [0.252, 0.395]**		**0.331 [0.268, 0.378]**	
State attachment anxiety → security	−0.006 [−0.037, 0.027]		−0.016 [−0.049, 0.017]	
*R*^2^ state attachment security	.278			
*R*^2^ state attachment anxiety	.234			
	State attachment security → anxiety		State attachment anxiety → security	
Between-person predictors	β_unstandardized_ [95% CrI]	β_standardized_ [95% CrI]	β_unstandardized_ [95% CrI]	β_standardized_ [95% CrI]
Trait attachment avoidance	0.064 [−0.030, 0.161]	0.133 [−0.061, 0.325]	−0.010 [−0.042, 0.023]	−0.075 [−0.302, 0.169]
Trait attachment anxiety	−0.024 [−0.101, 0.051]	−0.064 [−0.262, 0.140]	−0.004 [−0.030, 0.023]	−0.039 [−0.301, 0.206]
% time spent alone	0.022 [−0.529, 0.570]	0.009 [−0.213, 0.216]	0.029 [−0.153, 0.210]	0.042 [−0.212, 0.292]
Romantic relationship status	0.056 [−0.131, 0.247]	0.061 [−0.140, 0.253]	0.021 [−0.045, 0.089]	0.080 [−0.175, 0.317]
*R* ^2^	.057		.066	
Model 3: cross-lags between state attachment anxiety and avoidance				
Within-person effects	β_unstandardized_ [95% CrI]		β_standardized_ [95% CrI]	
State attachment anxiety → anxiety	**0.334 [0.255, 0.408]**		**0.349 [0.292, 0.390]**	
State attachment anxiety → avoidance	**−0.068 [−0.131, −0.006]**		**−0.086 [−0.134, −0.039]**	
State attachment avoidance → avoidance	**0.312 [0.234, 0.386]**		**0.295 [0.251, 0.347]**	
State attachment avoidance → anxiety	0.007 [−0.080, 0.096]		0.001 [−0.041, 0.046]	
*R*^2^ state attachment anxiety	.268			
*R*^2^ state attachment avoidance	.260			
	State attachment anxiety → avoidance		State attachment avoidance → anxiety	
Between-person predictors	β_unstandardized_ [95% CrI]	β_standardized_ [95% CrI]	β_unstandardized_ [95% CrI]	β_standardized_ [95% CrI]
Trait attachment avoidance	0.000 [−0.065, 0.067]	0.001 [−0.182, 0.196]	−0.026 [−0.113, 0.062]	−0.052 [−0.220, 0.125]
Trait attachment anxiety	0.008 [−0.042, 0.059]	0.032 [−0.161, 0.216]	0.061 [−0.003, 0.125]	0.163 [−0.009, 0.324]
% time spent alone	−0.110 [−0.453, 0.229]	−0.062 [−0.243, 0.130]	0.021 [−0.431, 0.487]	0.008 [−0.168, 0.185]
Romantic relationship status	−0.048 [−0.176, 0.074]	−0.073 [−0.250, 0.114]	0.086 [−0.074, 0.246]	0.091 [−0.077, 0.252]
*R* ^2^	.037		.060	

*Note. N*_participants_ = 122, *N*_observations_ = 4,629.

a Larger than the other cross-lagged effect based on the Wald test, p < .050. While the 95% credible interval (95% CrI) does not contain zero for the values in bold, the estimated standardized cross-lagged effects (the values in the right columns) between the state attachment dimensions were the averages of person-specific standardized coefficients in each sample (Schuurman et al., 2016). As a result, their 95% CrIs were a bit too underestimated, increasing the risk of Type I error (Schuurman et al., 2016). Therefore, the interpretation of whether the cross-lagged effects were detected was based on the unstandardized estimates (the values in the left columns). The results were summarized in R using the MplusAutomation package (Hallquist & Wiley, 2018).

**Table 3.** Sample II: Unstandardized and Standardized Cross-Lags Between State Attachment Dimensions and Associations of Trait Attachment with Cross-Lags.

Model 1: cross-lags between state attachment security and avoidance				
Within-person effects	β_unstandardized_ [95% CrI]		β_standardized_ [95% CrI]	
State attachment security → security	**0.334 [0.260, 0.405]**		**0.330 [0.293, 0.366]**	
State attachment security → avoidance	**−0.176 [−0.271, −0.056]** ^a^		**−0.120 [−0.166, −0.084]**	
State attachment avoidance → avoidance	**0.312 [0.245, 0.376]**		**0.315 [0.279, 0.349]**	
State attachment avoidance → security	−0.037 [−0.079, 0.004]		**−0.059 [−0.090, −0.027]**	
*R*^2^ state attachment security	.262			
*R*^2^ state attachment avoidance	.276			
	State attachment security → avoidance		State attachment avoidance → security	
Between-person predictors	β_unstandardized_ [95% CrI]	β_standardized_ [95% CrI]	β_unstandardized_ [95% CrI]	β_standardized_ [95% CrI]
Trait attachment avoidance	−0.007 [−0.124, 0.105]	−0.013 [−0.220, 0.203]	−0.025 [−0.060, 0.011]	−0.123 [−0.293, 0.051]
Trait attachment anxiety	0.053 [−0.028, 0.135]	0.121 [−0.062, 0.299]	−0.013 [−0.041, 0.014]	−0.077 [−0.230, 0.081]
% Time spent alone	0.150 [−0.461, 0.762]	0.038 [−0.110, 0.188]	0.029 [−0.212, 0.272]	0.019 [−0.134, 0.168]
Romantic relationship status	0.041 [−0.174, 0.254]	0.038 [−0.159, 0.225]	−0.057 [−0.133, 0.018]	−0.135 [−0.310, 0.043]
*R* ^2^	.045		.056	
Model 2: cross-lags between state attachment security and anxiety				
Within-person effects	β_unstandardized_ [95% CrI]		β_standardized_ [95% CrI]	
State attachment security → security	**0.339 [0.270, 0.404]**		**0.336 [0.296, 0.368]**	
State attachment security → anxiety	**−0.144 [−0.233, −0.055]** ^a^		**−0.071 [−0.105, −0.031]**	
State attachment anxiety → anxiety	**0.416 [0.359, 0.469]**		**0.413 [0.366, 0.452]**	
State attachment anxiety → security	−0.014 [−0.036, 0.007]		−0.023 [−0.051, 0.006]	
*R*^2^ state attachment security	.217			
*R*^2^ state attachment anxiety	.263			
	State attachment security → anxiety		State attachment anxiety → security	
Between-person predictors	β_unstandardized_ [95% CrI]	β_standardized_ [95% CrI]	β_unstandardized_ [95% CrI]	β_standardized_ [95% CrI]
Trait attachment avoidance	−0.023 [−0.114, 0.069]	−0.067 [−0.317, 0.191]	0.006 [−0.008, 0.020]	0.133 [−0.217, 0.404]
Trait attachment anxiety	−0.007 [−0.074, 0.060]	−0.025 [−0.241, 0.202]	−0.004 [−0.015, 0.007]	−0.109 [−0.398, 0.203]
% time spent alone	0.266 [−0.245, 0.773]	0.100 [−0.090, 0.278]	−0.024 [−0.132, 0.095]	−0.071 [−0.455, 0.271]
Romantic relationship status	−0.101 [−0.281, 0.076]	−0.142 [−0.363, 0.104]	−0.004 [−0.033, 0.024]	−0.047 [−0.394, 0.244]
*R* ^2^	.074		.124	
Model 3: cross-lags between state attachment anxiety and avoidance				
Within-person effects	β_unstandardized_ [95% CrI]		β_standardized_ [95% CrI]	
State attachment anxiety → anxiety	**0.434 [0.378, 0.486]**		**0.434 [0.401, 0.466]**	
State attachment anxiety → avoidance	0.024 [−0.013, 0.060]		**0.041 [0.003, 0.074]**	
State attachment avoidance → avoidance	**0.325 [0.256, 0.388]**		**0.327 [0.282, 0.363]**	
State attachment avoidance → anxiety	0.006 [−0.050, 0.061]		0.011 [−0.014, 0.042]	
*R*^2^ state attachment anxiety	.259			
*R*^2^ state attachment avoidance	.245			
	State attachment anxiety → avoidance		State attachment avoidance → anxiety	
Between-person predictors	β_unstandardized_ [95% CrI]	β_standardized_ [95% CrI]	β_unstandardized_ [95% CrI]	β_standardized_ [95% CrI]
Trait attachment avoidance	**0.048 [0.008, 0.090]**	**0.232 [0.037, 0.407]**	0.019 [−0.036, 0.070]	0.108 [−0.180, 0.408]
Trait attachment anxiety	**0.047 [0.018, 0.076]**	**0.266 [0.107, 0.401]**	0.024 [−0.014, 0.062]	0.161 [−0.089, 0.407]
% time spent alone	0.079 [−0.127, 0.286]	0.049 [−0.074, 0.185]	−0.268 [−0.561, 0.036]	−0.196 [−0.421, 0.025]
Romantic relationship status	**0.145 [0.070, 0.222]**	**0.335 [0.168, 0.480]**	−0.026 [−0.132, 0.077]	−0.070 [−0.342, 0.208]
*R* ^2^	.257		.137	

*Note. N*_participants_ = 127, *N*_observations_ = 5,322.

a Larger than the other cross-lagged effect based on the Wald test, *p* < .050. While the 95% credible interval (95% CrI) does not contain zero for the values in bold, the estimated standardized cross-lagged effects (the values in the right columns) between the state attachment dimensions were the averages of person-specific standardized coefficients in each sample (Schuurman et al., 2016). As a result, their 95% CrIs were a bit too underestimated, increasing the risk of Type I error (Schuurman et al., 2016). Therefore, the interpretation of whether the cross-lagged effects were detected was based on the unstandardized estimates (the values in the left columns). The results were summarized in *R* using the MplusAutomation package (Hallquist & Wiley, 2018).

### Within-Person State Attachment Cross-Lags

We detected several associations related to our first research aim on state attachment cross-lags and the second aim on their relative dominance. For state security and avoidance, in line with our hypothesis, high security (i.e., higher-than-average levels of security for an individual^7^) was related to a subsequent decrease in avoidance in both samples. These cross-lagged associations can be regarded as large relative to the existing empirical literature (Orth et al., 2024). Contrary to our hypotheses, no cross-lagged effects of avoidance on security were detected in Sample I or Sample II. Moreover, Wald tests and the descriptive comparisons of the 95% CrIs of the standardized coefficients (i.e., checking whether the CrI for one effect excludes the point estimate of the other) indicated that the effect of security on avoidance was larger than that of avoidance on security in Sample I, χ^2^ [1] = 5.531, *p* = .019, and in Sample II, χ^2^ [1] = 5.111, *p* = .024.

For state security and anxiety, in line with our hypothesis, high security was related to a subsequent decrease in anxiety in Sample II. This association can be considered medium in terms of the empirical benchmarks (Orth et al., 2024). Wald tests and the descriptive comparisons of 95% CrIs of the standardized effects suggested that the effect of security on anxiety was larger than that of anxiety on security, χ^2^ [1] = 7.588, *p* = .006. The latter effect was not even detected in Sample II, which contrasted with our hypothesis. Contrary to our hypotheses, no cross-lags between security and anxiety were detected in Sample I.

Finally, when exploring state anxiety-avoidance dynamics, high anxiety was related to a decrease in avoidance in Sample I. This association can be considered medium relative to empirical benchmarks (Orth et al., 2024). No cross-lagged effect of avoidance on anxiety was detected in Sample I. Although the Wald test was not significant in Sample I, χ^2^ [1] = 1.838, *p* = .175, the descriptive comparisons of 95% CrIs of the standardized effects indicated that the effect of anxiety on avoidance was larger than that of avoidance on anxiety (Table 2). However, in Sample II, no effects were detected between anxiety and avoidance.

### Between-Person Moderation Effects of Trait Attachment

Compared to our first and second research aims, the findings for our third aim, which explored the role of trait attachment in state attachment cross-lags, were more modest. Contrary to our hypotheses, no associations of trait attachment avoidance were detected with the cross-lags between state security and avoidance. Similarly, contrary to our hypotheses, no associations of trait attachment anxiety were detected with the cross-lags between state security and anxiety.

Nevertheless, in Sample II, two unexpected associations for trait attachment emerged. Both high trait attachment avoidance and anxiety amplified the cross-lagged effect of state anxiety on increased state avoidance. These moderation effects, illustrated in Figures S2A and S2B in Supplemental Material 2, indicated that the direction of cross-lagged effects varied depending on trait attachment levels. Specifically, among individuals with higher trait attachment avoidance and anxiety, state anxiety predicted increased state avoidance, whereas among those with lower trait attachment anxiety and avoidance levels, state anxiety predicted decreased state avoidance (Figures S2A and S2B). However, these patterns were not replicated in Sample I.

### Sensitivity Analyses

After completing our primary preregistered analyses, we conducted pooled, nonpreregistered sensitivity analyses by combining both samples to evaluate the robustness of our findings. These analyses were particularly relevant to our third research aim, which examined the role of trait attachment in moderating state attachment cross-lags. Our main analyses, along with additional power simulations for the smallest detectable effects (see next section), suggested that, within individual samples, the power to detect the moderation effects was modest, especially for smaller effect sizes.

Table 4 presents the results of the nonpreregistered sensitivity analyses for the pooled sample. These analyses replicated the cross-lagged effects of state security on decreased avoidance and anxiety. The pooled analyses also revealed that high state avoidance predicted a medium-sized decrease in security. This effect was not detected in individual sample analyses (Tables 2 and 3). Yet, in line with individual sample analyses, the effect of security on avoidance was again larger than that of avoidance on security, χ^2^ [1] = 6.158, *p* = .013. No cross-lagged effects were found between state anxiety and avoidance, which contrasted with Sample I results.

**Table 4.** Pooled Sample: Unstandardized and Standardized Cross-Lags Between State Attachment Dimension and Associations of Trait Attachment with Cross-Lags.

Model 1: cross-lags between state attachment security and avoidance				
Within-person effects	β_unstandardized_ [95% CrI]		β_standardized_ [95% CrI]	
State attachment security → security	**0.371 [0.322, 0.418]**		**0.369 [0.340, 0.400]**	
State attachment security → avoidance	**−0.152 [−0.222, −0.077]** ^a^		**−0.105 [−0.132, −0.081]**	
State attachment avoidance → avoidance	**0.310 [0.262, 0.356]**		**0.313 [0.283, 0.340]**	
State attachment avoidance → security	**−0.050 [−0.078, −0.021]**		**−0.071 [−0.098, −0.049]**	
*R*^2^ state attachment security	.282			
*R*^2^ state attachment avoidance	.261			
	State attachment security → avoidance		State attachment avoidance → security	
Between-person predictors	β_unstandardized_ [95% CrI]	β_standardized_ [95% CrI]	β_unstandardized_ [95% CrI]	β_standardized_ [95% CrI]
Trait attachment avoidance	−0.042 [−0.106, 0.019]	−0.094 [−0.224, 0.044]	−0.019 [−0.044, 0.007]	−0.097 [−0.222, 0.034]
Trait attachment anxiety	0.011 [−0.034, 0.058]	0.031 [−0.091, 0.160]	−0.018 [−0.038, 0.001]	−0.116 [−0.233, 0.007]
% Time spent alone	0.127 [−0.116, 0.369]	0.057 [−0.051, 0.169]	−0.085 [−0.199, 0.028]	−0.088 [−0.205, 0.028]
Romantic relationship status	−0.067 [−0.178, 0.049]	−0.075 [−0.200, 0.053]	**−0.052 [−0.104, −0.002]**	**−0.133 [−0.260, −0.005]**
*R* ^2^	.031		.059	
Model 2: cross-lags between state attachment security and anxiety				
Within-person effects	β_unstandardized_ [95% CrI]		β_standardized_ [95% CrI]	
State attachment security → security	**0.389 [0.344, 0.432]**		**0.389 [0.364, 0.411]**	
State attachment security → anxiety	**−0.069 [−0.134, −0.003]** ^a^		**−0.037 [−0.060, −0.012]**	
State attachment anxiety → anxiety	**0.397 [0.355, 0.436]**		**0.401 [0.370, 0.428]**	
State attachment anxiety → security	0.001 [−0.015, 0.016]		−0.005 [−0.028, 0.015]	
*R*^2^ state attachment security	.239			
*R*^2^ state attachment anxiety	.246			
	State attachment security → anxiety		State attachment anxiety → security	
Between-person predictors	β_unstandardized_ [95% CrI]	β_standardized_ [95% CrI]	β_unstandardized_ [95% CrI]	β_standardized_ [95% CrI]
Trait attachment avoidance	0.035 [−0.023, 0.094]	0.105 [−0.072, 0.267]	−0.002 [−0.015, 0.010]	−0.056 [−0.356, 0.248]
Trait attachment anxiety	−0.014 [−0.059, 0.032]	−0.050 [−0.210, 0.120]	−0.003 [−0.012, 0.007]	−0.079 [−0.341, 0.229]
% time spent alone	0.208 [−0.032, 0.447]	0.126 [−0.020, 0.267]	0.014 [−0.041, 0.062]	0.066 [−0.216, 0.361]
Romantic relationship status	0.039 [−0.067, 0.144]	0.059 [−0.099, 0.212]	−0.006 [−0.028, 0.017]	−0.078 [−0.357, 0.205]
*R* ^2^	.052		.091	
Model 3: cross-lags between state attachment anxiety and avoidance				
Within-person effects	β_unstandardized_ [95% CrI]		β_standardized_ [95% CrI]	
State attachment anxiety → anxiety	**0.414 [0.373, 0.452]**		**0.415 [0.389, 0.438]**	
State attachment anxiety → avoidance	−0.028 [−0.060, 0.002]		−0.024 [−0.049, 0.000]	
State attachment avoidance → avoidance	**0.325 [0.277, 0.371]**		**0.326 [0.293, 0.355]**	
State attachment avoidance → anxiety	0.007 [−0.037, 0.052]		0.007 [−0.020, 0.042]	
*R*^2^ state attachment anxiety	.252			
*R*^2^ state attachment avoidance	.240			
	State attachment anxiety → avoidance		State attachment avoidance → anxiety	
Between-person predictors	β_unstandardized_ [95% CrI]	β_standardized_ [95% CrI]	β_unstandardized_ [95% CrI]	β_standardized_ [95% CrI]
Trait attachment avoidance	0.023 [−0.014, 0.059]	0.102 [−0.058, 0.262]	0.004 [−0.041, 0.047]	0.017 [−0.170, 0.217]
Trait attachment anxiety	**0.029 [0.004, 0.056]**	**0.162 [0.020, 0.289]**	**0.038 [0.008, 0.069]**	**0.211 [0.042, 0.363]**
% time spent alone	−0.029 [−0.161, 0.099]	−0.027 [−0.145, 0.088]	−0.145 [−0.309, 0.021]	−0.132 [−0.288, 0.019]
Romantic relationship status	**0.073 [0.008, 0.137]**	**0.163 [0.017, 0.299]**	0.016 [−0.061, 0.090]	0.036 [−0.132, 0.203]
*R* ^2^	.078		.086	

*Note. N*_participants_ = 249, *N*_observations_ = 9,951.

a Larger than the other cross-lagged effect based on the Wald test, *p* < .050. While the 95% credible interval (95% CrI) does not contain zero for the values in bold, the estimated standardized cross-lagged effects (the values in the right columns) between the state attachment dimensions were the averages of person-specific standardized coefficients in each sample (Schuurman et al., 2016). As a result, their 95% CrIs were a bit too underestimated, increasing the risk of Type I error (Schuurman et al., 2016). Therefore, the interpretation of whether the cross-lagged effects were detected was based on the unstandardized estimates (the values in the left columns). The results were summarized in R using the MplusAutomation package (Hallquist & Wiley, 2018).

Consistent with the findings from Sample II, trait attachment anxiety moderated the cross-lagged effect of state anxiety on state avoidance. It also moderated the effect of state avoidance on state anxiety. Again, the crossover nature of these moderation effects appeared to be present: Among individuals with higher trait attachment anxiety, state anxiety, and avoidance reinforced each other, whereas among those with lower trait attachment anxiety, they predicted decreases in each other (see Figures S2C and S2D in Supplemental Material 2). Trait attachment avoidance did not moderate the effect of state anxiety on avoidance, contrasting with Sample II results.

Supplemental Material 3 contains the preregistered sensitivity analyses where state attachment at the previous assessment was treated as the lagged observation of state attachment at the current assessment without considering the differences in their time lags. Most interpretations remain the same regarding the cross-lagged effects. Similar to pooled analyses, only trait attachment anxiety (not avoidance) strengthened the effect of state attachment anxiety on increased avoidance in Sample II.

Supplemental Material 4 displays the preregistered sensitivity analyses where all three state dimensions at the previous moment were used to predict one state attachment dimension at the current moment. All cross-lagged effects detected in our main models were replicated between state attachment dimensions. Moreover, as in the pooled analyses, state attachment avoidance predicted decreased security in both samples.

Finally, in the nonpreregistered models where all cross-lags were modeled simultaneously, convergence was achieved in Sample II but not in Sample I or the pooled sample. The results mirrored our primary models, replicating the main findings for Sample II: state security predicted decreased avoidance (β = −.189, 95% CrI [−0.263, −0.116], β* = −.139) and anxiety (β = −.112, 95% CrI [−0.180, −0.047], β* = −.066).

### Power Simulations

Lastly, we executed Monte Carlo simulations to evaluate the power concerning the detection of the smallest effect sizes. At the within-person level, our design incorporated a substantial volume of observations for both samples. Therefore, our focus in the simulations was to assess the capability of our design to identify the moderating effects of trait attachment.

Upon conducting 500 replications, our simulations indicated that the standardized effects required to achieve a power exceeding 0.80 for the effect of trait attachment dimensions on the cross-lags were in the ranges of |0.280| to |0.410| for Sample I. For Sample II, due to smaller random effect estimates for the cross-lags, the standardized effects exceeding the power of 0.80 were larger, ranging from |0.370| to |0.600|. Finally, for the pooled sample, the standardized effects exceeding power of .80 were in the ranges of |0.250| to |0.440|. Thus, while Sample II was sufficient to detect only relatively large moderation effects of trait attachment on state attachment cross-lags, the power in the analyses for Sample I and the pooled sample was adequate to detect medium-sized effects.

## Discussion

**Figure 3:**
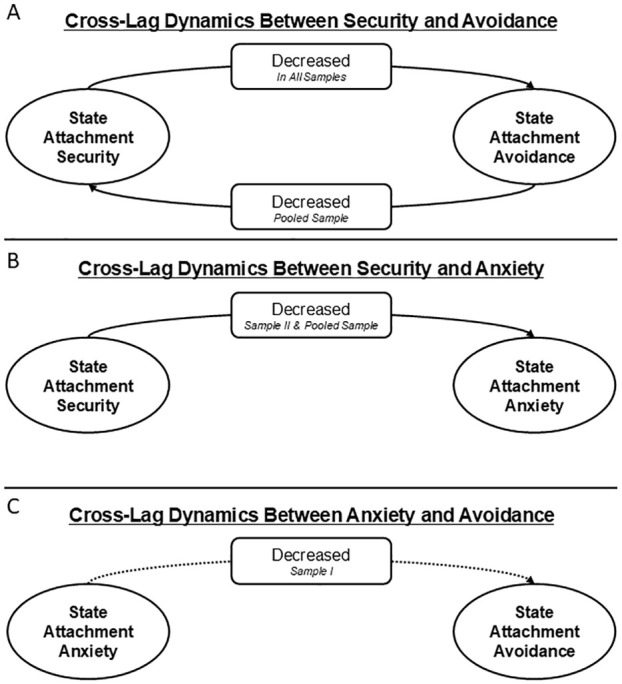
Summary of findings: detected state attachment cross-lags between (A) security and avoidance, (B) security and anxiety, and (C) anxiety and avoidance. *Note.* Solid arrows represent hypothesized associations, while rounded dotted arrows refer to associations not hypothesized.

The attachment system motivates individuals to seek and maintain a sense of security and cope with insecurities when security is out of reach. These processes necessitate the dynamic coordination of various attachment states; however, research in this area has remained scant. In this two-sample EMA study, we sought new insights into the everyday functioning of the attachment system by examining the cross-lagged effects between state attachment dimensions. Figure 3 summarizes the main findings, showing that while some patterns were robustly replicated, others exhibited greater variability across samples.

Our primary findings were that state security predicted decreased avoidance in both samples and decreased anxiety in Sample II. These findings corroborated our hypotheses and were further replicated in sensitivity analyses of the pooled sample. Additionally, although state avoidance did not show cross-lagged effects in the individual sample analyses, it predicted decreased security in the pooled sample, aligning with our hypothesis. Finally, although it was not hypothesized, state anxiety predicted decreased avoidance in Sample I (but not in Sample II or the pooled sample). Beyond testing the average state attachment cross-lagged effects, our initial steps to consider whether and how trait attachment moderates these cross-lags provided no support for our hypotheses. Yet, some unexpected moderation effects emerged in one of the samples as well as in pooled analyses. In essence, our findings underscore the predominant role of state security in coordinating everyday attachment dynamics.

### Role of State Attachment Security in Coordinating Everyday Attachment Dynamics

The primary discovery of our study was the cross-lagged effects of state security on changes in avoidance and anxiety. Specifically, in line with our hypotheses, we found that state security was linked to reduced state avoidance and anxiety. These findings are in accord with theoretical models of the attachment system, which propose that the system’s set goal is to maintain and attain security, whereas hyperactivation and deactivation are secondary strategies to cope with insecurities (Long et al., 2020; Mikulincer & Shaver, 2016, 2023). Thus, our primary findings may reflect the attachment system’s inherent drive to sustain stability and resist change when feeling secure (Long et al., 2020; Mikulincer & Shaver, 2016, 2023). Alternatively, they may indicate that low security tends to activate increased anxiety or avoidance when the primary strategy to attain security is not viable (Long et al., 2020; Mikulincer & Shaver, 2016, 2023).

Importantly, we observed no hypothesized cross-lagged effects of state avoidance or anxiety on reduced security in our primary analyses for individual samples. Further, the comparisons of cross-lagged effects consistently revealed the relative dominance of security over both avoidance and anxiety. This asymmetry underscores the centrality of state security in everyday attachment dynamics. Specifically, it suggests that the attachment system module responsible for appraising the success of the primary attachment strategy (i.e., seeking an actual or symbolic source of security) provides crucial information for evaluating the viability of secondary hyperactivation and deactivation strategies (Mikulincer & Shaver, 2016, 2023). Thus, fluctuations in the sense of security appear to play a gatekeeping role in coordinating other attachment responses, while avoidance and anxiety may contribute relatively less feedback to shaping that sense of security.

Nonetheless, in the sensitivity analyses of the pooled sample, we found a hypothesized effect of state avoidance predicting decreased state security. This finding may indicate deactivation processes that aim to limit attachment-related information from entering awareness (Bowlby, 1980). As noted in previous research, such a defensive approach may help individuals avoid the psychological pain stemming from the experience of being rejected (Dykas & Cassidy, 2011). Alternatively, state avoidance may be accompanied by distancing interpersonal behaviors that preclude social connection and lower the sense of security (Kaurin et al., 2022; Tammilehto et al., 2023). Interestingly, in the pooled sample, we observed this cross-lagged effect on decreased security only for avoidance, but not anxiety. This discrepancy may suggest that state anxiety might, at times, help individuals restore a sense of security in the context of daily life. However, regaining security may become significantly more challenging in more threatening daily situations involving strong uncertainty about an attachment figure’s availability (Arriaga et al., 2018). To gain a more precise understanding of the differences between states of anxiety and avoidance, future EMA research would benefit from considering situational factors (e.g., the degree of cues about an attachment figure’s availability) that can shape the consequences of the different strategies.

Finally, in Sample I, we detected a cross-lagged effect of state anxiety on decreased avoidance, but not vice versa. While not hypothesized, this exploratory finding further aligns with the function of state anxiety to foster proximity to the attachment figure (Gillath et al., 2009; Tammilehto et al., 2022). More specifically, it suggests that individuals’ momentary hyperactivation motivation (i.e., profound yearning for intimacy and affirmation) may override the deactivation motivation (i.e., shunning reliance on unresponsive attachment figures). This corroborates with the theoretical models underscoring the functionally separate strategies to deal with different forms of insecurities (Arriaga et al., 2018; Long et al., 2020; Mikulincer & Shaver, 2016, 2023). However, the lack of replication across samples stresses the need for further research to confirm the robustness of this exploratory finding.

### No Evidence for Expected Role of Trait Attachment in Cross-Lags

Regarding the moderating role of trait attachment, we found no support for our original hypotheses. First, we found no evidence for the hypothesized role of high trait avoidance in amplifying the effect of state avoidance on diminished security and weakening the effect of security in lowering avoidance (Arriaga et al., 2018; Bosmans et al., 2020). Second, we found no evidence supporting the corresponding role of trait anxiety in moderating the dynamics between state anxiety and security (Arriaga et al., 2018; Bosmans et al., 2020).

Caution is warranted when interpreting these findings. While our study provides a valuable starting point for understanding the role of trait attachment in state attachment cross-lags, our power simulations indicated that we were only able to detect medium-to-large moderation effects, increasing uncertainty around our null findings. Nevertheless, the absence of evidence for our hypotheses aligns with a recent large-scale meta-analysis that found no robust support for the moderating role of trait attachment in security priming outcomes (Gillath et al., 2022). It also mirrors our other recent study, which showed that trait attachment dimensions were only linked with baseline levels and variability of state attachment dimensions, but no associations were found with autoregressive dynamics (i.e., inertia; Tammilehto, Kaurin, et al., 2025). Taken together, these findings suggest that trait attachment might primarily manifest in the average levels and variability of state attachment rather than in how state attachment dimensions persist and influence each other in daily life. Yet, future research with larger EMA samples is needed to further clarify the role of trait attachment in temporal dynamics of state attachment.

Although our hypotheses were not supported, we observed a noteworthy nonhypothesized pattern: high trait attachment anxiety amplified (a) the cross-lagged effect of state anxiety on increased state avoidance in Sample II and the pooled sample, and (b) the cross-lagged effect of state avoidance on increased state anxiety in the pooled sample. These bidirectional associations between insecure states among individuals with high trait attachment anxiety may indicate that heightened state avoidance reflects their ambivalent feelings of anger and frustration toward unavailable attachment figures, rather than genuine efforts to deactivate and dismiss attachment needs (Bowlby, 1973). Alternatively, the bidirectional links may represent the escalation of insecure attachment dynamics, where hyperactivation and deactivation motivations amplify each other chaotically and indiscriminately (Mikulincer & Shaver, 2016). In contrast, among individuals with lower trait attachment anxiety, our findings suggest a resiliency pattern in which high levels of one insecure state do not spill over into the other. However, extra caution is warranted when interpreting the cross-over interactions, as the ECR-R has shown lower discriminability among individuals scoring at the lower end of the trait dimensions (Fraley et al., 2000). Overall, given the nonhypothesized nature of these preliminary findings, future research is needed to confirm their robustness.

Finally, the least robust cross-over moderation effect—observed only in Sample II—indicated that higher trait attachment avoidance amplified the cross-lagged effect of state anxiety on increased avoidance. In contrast, lower trait attachment avoidance strengthened the cross-lagged effect of state anxiety on decreased state avoidance. Although preliminary and in need of confirmation through future research, this cross-over moderation is notable as it occurs in the same direction as that observed for trait attachment anxiety. While we proposed above that the similar amplification associated with high trait anxiety might reflect ambivalent anger rather than a genuine deactivation effort, the moderation effect of high trait avoidance does align with attachment theory’s account of deactivation. Specifically, the activation of attachment needs for closeness and protection may prompt individuals higher trait attachment avoidance to suppress these needs through deactivation efforts (Bowlby, 1980; Mikulincer & Shaver, 2023). Thus, they may respond to heightened state anxiety by intensifying their state avoidance.

### General Discussion

On the whole, our study serves as the first in vivo demonstration of the sequential modules and processes of the attachment system related to security and insecurities in everyday life. It underscores the dominant role of state security in attachment dynamics. This theoretical insight resonates closely with the broaden-and-build model of attachment security (Mikulincer & Shaver, 2016, 2020, 2023). This model asserts that a sense of security enhances a person’s resilience, improving their ability to cope, access resources, and adapt flexibly (Mikulincer & Shaver, 2016, 2020, 2023). Interestingly, several other recent EMA studies have also underscored the significance of fluctuations in security over avoidance and anxiety when it comes to guiding emotion regulation (Tammilehto et al., 2022) and interpersonal dynamics (Kaurin et al., 2022). This convergence of EMA evidence consistently suggests that the foundation of the attachment system may be deeply rooted in the general sense of security (Long et al., 2020; Mikulincer & Shaver, 2016, 2023). In turn, avoidance and anxiety emerge as secondary processes that follow the initial appraisals related to a lack of security (Long et al., 2020; Mikulincer & Shaver, 2016, 2023).

In addition to its theoretical significance, the observed centrality of security in coordinating attachment dynamics highlights the potential of security-enhancing interventions to improve socioemotional well-being (Slade & Holmes, 2019). Achieving and maintaining security through processes that encourage positive representations of self and others may reduce and prevent insecure states (Arriaga et al., 2018). These processes focus on (a) confidence-building, such as promoting autonomy and self-efficacy during challenges, and (b) positive dependence, such as rewarding interdependent activities and trust in others without eliciting resistance (Arriaga et al., 2018). However, further research is essential to unravel the intra- and interpersonal mechanisms that strengthen individuals’ sense of security in their daily lives. Refining our understanding of these processes could lead to improved psychological tools for enhancing socioemotional well-being and relationships.

### Strengths and Limitations

Our study provided a first comprehensive examination of everyday state attachment cross-lags, potentially sparking further interest in this emerging area of state attachment research. We effectively modeled state attachment dynamics using two independent EMA samples with a preregistered set of hypotheses and an analytical plan. This offered a robust and transparent design for scrutinizing state attachment cross-lags from one moment to the next.

However, our study has some limitations that should be acknowledged. First, as already discussed, while our design was sufficiently robust to detect the within-person associations between state attachment dimensions, it possessed more limited power to discern the between-person moderating effects of trait attachment. Larger sample sizes in the future could offer a more nuanced understanding of the role of trait attachment in state attachment cross-lags. Second, the overrepresentation of females and the focus on Western participants limit the generalization of our findings to males and non-Western populations. Third, although all reliabilities met EMA benchmarks (Nezlek, 2017), state anxiety showed relatively lower within-person reliability. Future EMA studies should scrutinize the number of items necessary to assess state attachment anxiety reliably. While more items may enhance reliability, this may also amplify the burden for participants, leading to less accurate responses (Eisele et al., 2022). Therefore, a delicate balance must be maintained. Fourth and related to the previous point, a key consideration overlooked in our modeling approach was the potential impact of measurement error, which could have affected our cross-lagged estimates (Driver, 2025; Schuurman & Hamaker, 2019). In future research employing designs with a larger number of EMA observations, researchers might consider utilizing DSEM-based methods developed to account for measurement errors (Schuurman & Hamaker, 2019). Fifth, the extent to which our trait-level findings generalize to research frameworks that assess attachment using the Adult Attachment Interview (AAI) remains uncertain. While AAI exhibits only small correlations with self-report attachment measures (Roisman et al., 2007), both methods have been consistently linked to theoretically meaningful outcomes (Hesse, 2016; X. Zhang et al., 2022). This stresses the importance of using multimethod approach in future studies, as different methods may capture complementary aspects of attachment. Lastly, our findings on state attachment cross-lags should be interpreted as applying only to 1-hr lagged windows, given our use of discrete-time models. Future research would benefit from using continuous-time approaches to yield more causally informative insights into state attachment cross-lags (Driver & Voelkle, 2018; Tammilehto, Saarni, et al., 2025).

## Conclusions

The exploration of changes in attachment states represents a novel focus in attachment research with significant theoretical and therapeutic implications. Our EMA study with two independent samples contributed to this discourse by examining the short-term dynamics between state attachment security, avoidance, and anxiety in natural settings. Our findings emphasize the dominance of state attachment security over avoidance and anxiety in coordinating the temporal flow of daily attachment experiences. This suggests that the everyday functioning of the attachment system may be deeply embedded in fluctuations in the general sense of security. We hope our study will inspire further research to uncover the processes and mechanisms that strengthen individuals’ sense of security. Such research can advance our ability to enhance socioemotional well-being and improve intimate relationships.

## Supplemental Material

sj-docx-1-psp-10.1177_01461672251333472 – Supplemental material for Temporal Dynamics Between State Attachment Security, Avoidance, and Anxiety: Insights into Everyday Attachment System Functioning Supplemental material, sj-docx-1-psp-10.1177_01461672251333472 for Temporal Dynamics Between State Attachment Security, Avoidance, and Anxiety: Insights into Everyday Attachment System Functioning by Jaakko Tammilehto, Aleksandra Kaurin, Peter Kuppens, Guy Bosmans, Mervi Vänskä, Marjo Flykt, Kirsi Peltonen and Jallu Lindblom in Personality and Social Psychology Bulletin

sj-docx-2-psp-10.1177_01461672251333472 – Supplemental material for Temporal Dynamics Between State Attachment Security, Avoidance, and Anxiety: Insights into Everyday Attachment System Functioning Supplemental material, sj-docx-2-psp-10.1177_01461672251333472 for Temporal Dynamics Between State Attachment Security, Avoidance, and Anxiety: Insights into Everyday Attachment System Functioning by Jaakko Tammilehto, Aleksandra Kaurin, Peter Kuppens, Guy Bosmans, Mervi Vänskä, Marjo Flykt, Kirsi Peltonen and Jallu Lindblom in Personality and Social Psychology Bulletin

sj-docx-3-psp-10.1177_01461672251333472 – Supplemental material for Temporal Dynamics Between State Attachment Security, Avoidance, and Anxiety: Insights into Everyday Attachment System Functioning Supplemental material, sj-docx-3-psp-10.1177_01461672251333472 for Temporal Dynamics Between State Attachment Security, Avoidance, and Anxiety: Insights into Everyday Attachment System Functioning by Jaakko Tammilehto, Aleksandra Kaurin, Peter Kuppens, Guy Bosmans, Mervi Vänskä, Marjo Flykt, Kirsi Peltonen and Jallu Lindblom in Personality and Social Psychology Bulletin

sj-docx-4-psp-10.1177_01461672251333472 – Supplemental material for Temporal Dynamics Between State Attachment Security, Avoidance, and Anxiety: Insights into Everyday Attachment System Functioning Supplemental material, sj-docx-4-psp-10.1177_01461672251333472 for Temporal Dynamics Between State Attachment Security, Avoidance, and Anxiety: Insights into Everyday Attachment System Functioning by Jaakko Tammilehto, Aleksandra Kaurin, Peter Kuppens, Guy Bosmans, Mervi Vänskä, Marjo Flykt, Kirsi Peltonen and Jallu Lindblom in Personality and Social Psychology Bulletin

## Notes

1. The data for Sample I and its codebook are available at https://osf.io/3jhvt. The data for Sample II is not shared to protect the privacy of participants in the ongoing longitudinal study.

2.
https://osf.io/jr3ah/

3. All analysis scripts are available at https://osf.io/dzh4m.

4. More specifically, these results are comprehensively reported in our other study focusing on the univariate dynamic features of each state attachment dimension (Tammilehto, Kaurin, et al., 2025). Notably, that study was preregistered simultaneously with the current study. See also Tammilehto et al. (2022) regarding Sample I.

5. It should be noted that the estimated standardized cross-lagged effects between the state attachment dimensions were the average of person-specific standardized coefficients in each sample (Schuurman et al., 2016). As a result, their 95% CrIs were a bit too small, increasing the risk of type 1 error (Schuurman et al., 2016). Therefore, the interpretation of whether the cross-lagged effects were detected was based on the unstandardized estimates. Yet, together with Wald tests for unstandardized effects, we still used the 95% CrIs to compare the relative dominance of the standardized cross-lagged effects, as this is the best approach available (Schuurman et al., 2016).

6. Regarding the cross-lags, we estimated both fixed and random effects. Moreover, we estimated the fixed and random first-order autoregressive effects of security, avoidance, and anxiety and the fixed and random innovation variances and intercepts of each state attachment dimension.

7. The same interpretative logic applies to all the cross-lagged effects described.
