# Supplementary material for: Temporal Dynamics Between State Attachment Security, Avoidance, and Anxiety: Insights into Everyday Attachment System Functioning
Source: Pers Soc Psychol Bull. 2025 May 21;52(8):2382–98. doi: 10.1177/01461672251333472 (PMC13310265; doi:10.1177/01461672251333472)
Supplement: sj-docx-1-psp-10.1177_01461672251333472 – Supplemental material for Temporal Dynamics Between State Attachment Security, Avoidance, and Anxiety: Insights into Everyday Attachment System Functioning [file sj-docx-1-psp-10.1177_01461672251333472.docx]

**Supplemental Material 1: Mathematical Equations for Primary Dynamic Structural Equation Models**

Here, we present mathematical equations for our primary dynamic structural equation model (DSEM) depicted in Figure 2 of the actual research article. In this bivariate DSEM, the variance in state attachment (SA) dimensions was decomposed into within-person and between-person components (Hamaker et al., 2018, 2023):

$$SA I= \mu_{1i}+ {SA I}_{time,i}^{\left( W \right)}$$

$$SA II= \mu_{2i}+ {SA II}_{time,i}^{\left( W \right)}$$

where the $\mu_{1i}$ and $\mu_{2i}$ represent the within-person means, which form the between-person component of the model, and ${SA I}_{time,i}^{\left( W \right)}$ and ${SA II}_{time,i}^{\left( W \right)}$ capture the temporal deviations of individual 𝑖 at occasion *time* from these within-person means (Hamaker et al., 2018, 2023).

At the within-person level, the model can be expressed as (Hamaker et al., 2018, 2023):

$${SA I}_{time,i}^{\left( W \right)}= \varphi_{1,1i}{SA I}_{time-1,i}^{\left( W \right)}+ \varphi_{1,2i}{SA II}_{time-1,i}^{\left( W \right)}+{\sigma^{2}}_{1,i}$$

$${SA II}_{time,i}^{\left( W \right)}= \varphi_{2,1i}{SA I}_{time-1,i}^{\left( W \right)}+ \varphi_{2,2i}{SA II}_{time-1,i}^{\left( W \right)}+{\sigma^{2}}_{2,i}$$

Thus, we modeled the autoregressive effects (i.e., $\varphi_{1,1i}$ and $\varphi_{2,2i}$), cross-lagged effects (i.e., $\varphi_{2,1i}$ and $\varphi_{1,2i}$), and innovation variances (i.e., ${\sigma^{2}}_{1i}$ and ${\sigma^{2}}_{2i}$) for both dimensions (Hamaker et al., 2018, 2023).

At the between-person level, the model can be expressed as (Hamaker et al., 2018, 2023):

$$\mu_{1i}= \gamma_{SA I, 0}+ \mu_{SA I, 0,i}$$

$$\mu_{2i}= \gamma_{SA II, 0}+ \mu_{SA II, 0,i}$$

$$\varphi_{1,1i}= \gamma_{SA I, 1}+ \mu_{SA I, 1,i}$$

$$\varphi_{2,2i}= \gamma_{SA II, 1}+ \mu_{SA II, 1,i}$$

$$\varphi_{1,2i}= \gamma_{SA I, 2}+ \beta_{SAI21}trait anxiety+\beta_{SAI22}trait avoidance+\beta_{SAI23}\% being alone+\beta_{SAI24}relationship status+\mu_{SA I, 2,i}$$

$$\varphi_{2,1i}= \gamma_{SA II, 2}+ \beta_{SAII21}trait anxiety+\beta_{SAII22}trait avoidance+\beta_{SAII23}\% being alone+\beta_{SAII24}relationship status+ \mu_{SA II, 2,i}$$

$$Log\left( {\sigma^{2}}_{1,i} \right)=\gamma_{SA I, 3}+\mu_{SA I, 3,i}$$

$${{Log(\sigma}^{2}}_{2,i})= \gamma_{SA II, 3}+ \mu_{SA II, 3,i}$$

where the γ symbols represent the average (i.e., fixed) effects, and the $\mu$ terms denote individual deviations from these means (i.e., random effects). The β symbols, in turn, refer to the predictive effects of between-person covariates on the random components of the cross-lagged effects (Hamaker et al., 2018, 2023). Note that all between-person covariates were grand-mean centered.

Finally, the random effects are allowed to correlate at the between-person level. This can be expressed as (Hamaker et al., 2023):

$\begin{matrix} \begin{matrix} \begin{matrix} \begin{matrix} \begin{matrix} \begin{matrix} \mu_{SA I, 0,i} \\ \mu_{SA II, 0,i} \\ \mu_{SA I, 1,i} \end{matrix} \\ \mu_{SA II, 1,i} \end{matrix} \\ \mu_{SA I, 2,i} \end{matrix} \\ \mu_{SA II, 2,i} \end{matrix} \\ \mu_{SA I, 3,i} \end{matrix} \\ \mu_{SA II, 3,i} \end{matrix}$ ~ MN $\left( \left[ \begin{matrix} \begin{matrix} \begin{matrix} \begin{matrix} \begin{matrix} \begin{matrix} 0 \\ 0 \\ 0 \end{matrix} \\ 0 \end{matrix} \\ 0 \end{matrix} \\ 0 \end{matrix} \\ 0 \end{matrix} \\ 0 \end{matrix} \right],\left[ \begin{matrix} \tau_{SAI,0}^{2} & & & & & & & \\ \tau_{SAI,0; SAII,0} & \tau_{SAII,0}^{2} & & & & & & \\ \tau_{SAI,0; SAI,1} & \tau_{SAII,0; SAI,1} & \tau_{SAI,1}^{2} & & & & & \\ \tau_{SAI,0; SAII,1} & \tau_{SAII,0; SAII,1} & \tau_{SAI,1; SAII,1} & \tau_{SAII,1}^{2} & & & & \\ \tau_{SAI,0; SAI,2} & \tau_{SAII,0; SAI,2} & \tau_{SAI,1; SAI,2} & \tau_{SAII,1; SAI,2} & \tau_{SAI,2}^{2} & & & \\ \tau_{SAI,0; SAII,2} & \tau_{SAII,0; SAII,2} & \tau_{SAI,1; SAII,2} & \tau_{SAII,1; SAII,2} & \tau_{SAI,2; SAII,2} & \tau_{SAII,2}^{2} & & \\ \tau_{SAI,0; SAI,3} & \tau_{SAII,0; SAI,3} & \tau_{SAI,1; SAI,3} & \tau_{SAII,1; SAI,3} & \tau_{SAI,2; SAI,3} & \tau_{SAI,2; SAI,3} & \tau_{SAI,3}^{2} & \\ \tau_{SAI,0; SAII,3} & \tau_{SAII,0; SAII,3} & \tau_{SAI,1; SAII,3} & \tau_{SAII,1; SAII,3} & \tau_{SAI,2; SAII,3} & \tau_{SAI,2; SAII,3} & \tau_{SAI,3; SAII,3} & \tau_{SAII,3}^{2} \end{matrix} \right] \right)$

Thus, the random effects are assumed to come from a multivariate normal distribution, where the diagonal elements of the covariance matrix represent variances, and the off-diagonal elements represent covariances (Hamaker et al., 2023).

**References**

Hamaker, E. L., Asparouhov, T., Brose, A., Schmiedek, F., & Muthén, B. (2018). At the frontiers of modeling intensive longitudinal data: Dynamic structural equation models for the affective measurements from the COGITO study. *Multivariate Behavioral Research*, *53*(6), 820–841. https://doi.org/10.1080/00273171.2018.1446819

Hamaker, E. L., Asparouhov, T., & Muthen, B. (2023). Dynamic structural equation modeling as a combination of time series modeling, multilevel modeling, and structural equation modeling. In R. H. Hoyle, *The Handbook of Structural Equation Modeling* (2nd ed.). Guilford Press.
