# Supplementary material for: Temporal Dynamics Between State Attachment Security, Avoidance, and Anxiety: Insights into Everyday Attachment System Functioning
Source: Pers Soc Psychol Bull. 2025 May 21;52(8):2382–98. doi: 10.1177/01461672251333472 (PMC13310265; doi:10.1177/01461672251333472)
Supplement: sj-docx-2-psp-10.1177_01461672251333472 – Supplemental material for Temporal Dynamics Between State Attachment Security, Avoidance, and Anxiety: Insights into Everyday Attachment System Functioning [file sj-docx-2-psp-10.1177_01461672251333472.docx]

**Supplemental Material 2: Visualization of the Moderation Effects of Trait Attachment on State Attachment Cross-Lags**

**Figure S2A.** Moderation Role of Trait Attachment Anxiety in the Cross-Lagged Effect of State Attachment Anxiety on State Attachment Avoidance in Sample II

*
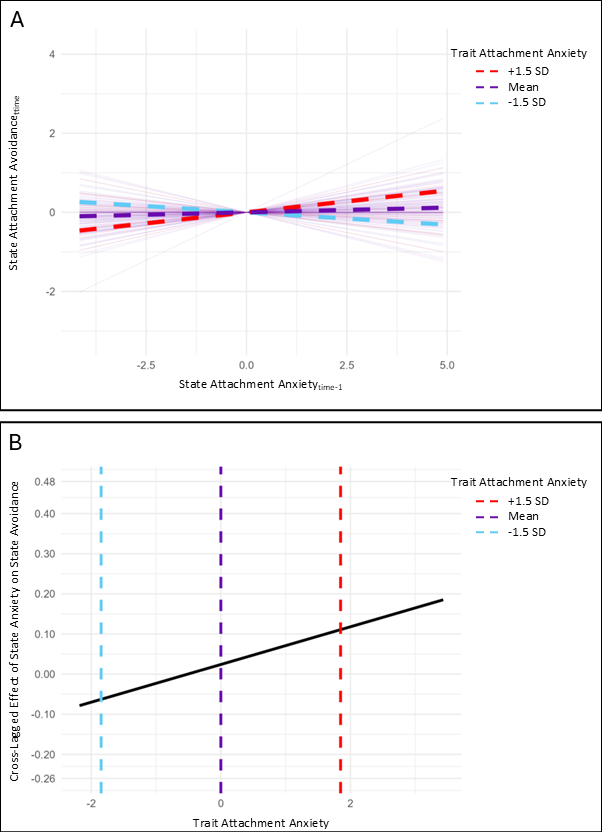
*

*Notes.* Panel A displays individual slopes derived from random slopes, along with fixed slopes corresponding to different levels of trait attachment anxiety. Panel B illustrates the predicted cross-lagged effect of state anxiety on state avoidance across varying levels of trait attachment anxiety. The red, purple, and blue dashed lines represent trait attachment anxiety levels at +1.5 SD, the mean, and -1.5 SD, respectively.

**Figure S2B.** Moderation Role of Trait Attachment Avoidance in the Cross-Lagged Effect of State Attachment Anxiety on State Attachment Avoidance in Sample II


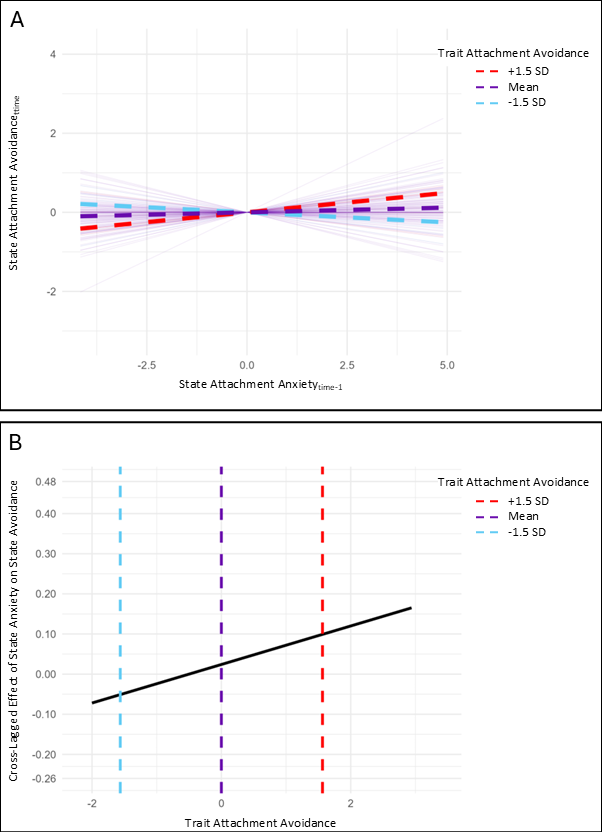


*Notes.* Panel A displays individual slopes derived from random slopes, along with fixed slopes corresponding to different levels of trait attachment avoidance. Panel B illustrates the predicted cross-lagged effect of state anxiety on state avoidance across varying levels of trait attachment avoidance. The red, purple, and blue dashed lines represent trait attachment avoidance levels at +1.5 SD, the mean, and -1.5 SD, respectively.

**Figure S2C.** Moderation Role of Trait Attachment Anxiety in the Cross-Lagged Effect of State Attachment Anxiety on State Attachment Avoidance in the Pooled Sample


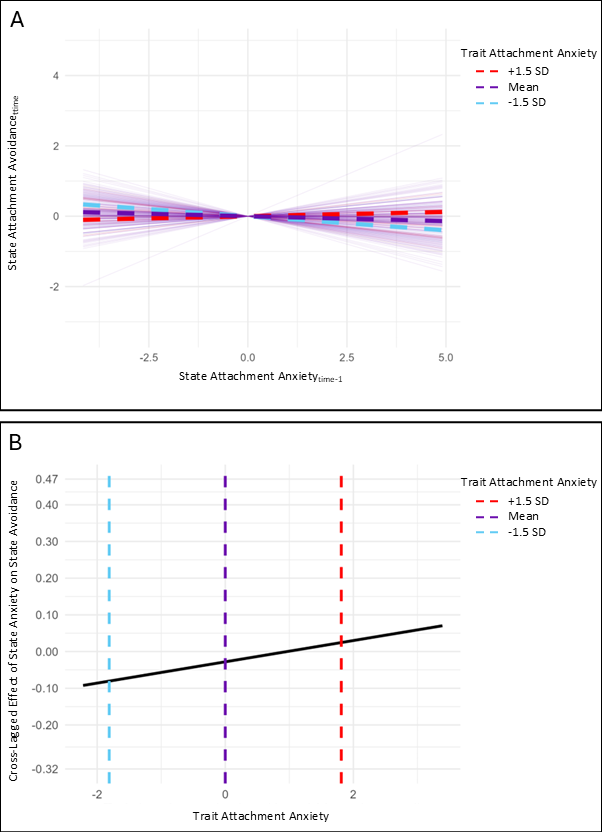


*Notes.* Panel A displays individual slopes derived from random slopes, along with fixed slopes corresponding to different levels of trait attachment anxiety. Panel B illustrates the predicted cross-lagged effect of state anxiety on state avoidance across varying levels of trait attachment anxiety. The red, purple, and blue dashed lines represent trait attachment anxiety levels at +1.5 SD, the mean, and -1.5 SD, respectively.

**Figure S2D.** Moderation Role of Trait Attachment Anxiety in the Cross-Lagged Effect of State Attachment Avoidance on State Attachment Anxiety in the Pooled Sample


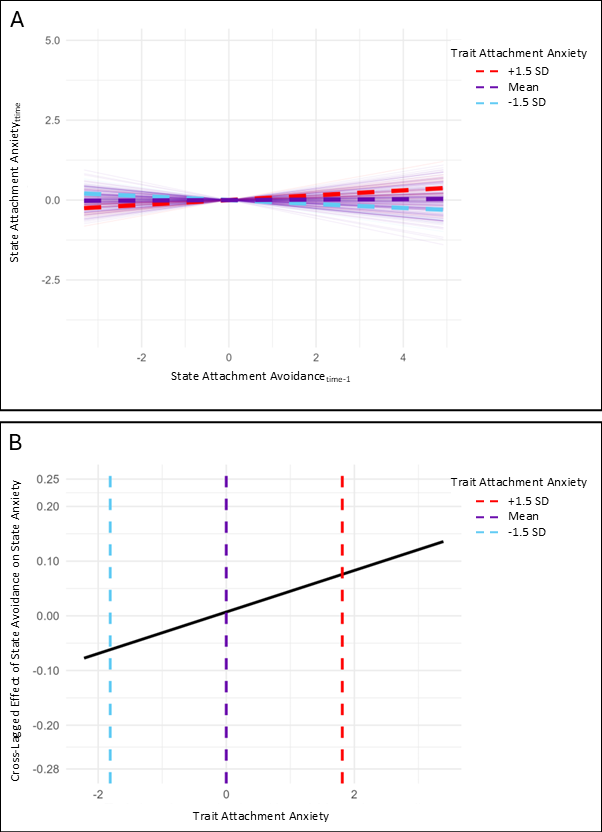


*Notes.* Panel A displays individual slopes derived from random slopes, along with fixed slopes corresponding to different levels of trait attachment anxiety. Panel B illustrates the predicted cross-lagged effect of state avoidance on state anxiety across varying levels of trait attachment anxiety. The red, purple, and blue dashed lines represent trait attachment anxiety levels at +1.5 SD, the mean, and -1.5 SD, respectively.
