# Supplementary material for: Temporal Dynamics Between State Attachment Security, Avoidance, and Anxiety: Insights into Everyday Attachment System Functioning
Source: Pers Soc Psychol Bull. 2025 May 21;52(8):2382–98. doi: 10.1177/01461672251333472 (PMC13310265; doi:10.1177/01461672251333472)
Supplement: sj-docx-3-psp-10.1177_01461672251333472 – Supplemental material for Temporal Dynamics Between State Attachment Security, Avoidance, and Anxiety: Insights into Everyday Attachment System Functioning [file sj-docx-3-psp-10.1177_01461672251333472.docx]

**Supplemental Material 3: Preregistered Sensitivity Analysis I: State Attachment at Previous Ecological Momentary Assessment as Lagged Observation**

| **Table S1A**  Sensitivity Analysis I for Sample I: Unstandardized and Standardized Cross-Lags Between State Attachment Dimensions and Associations of Trait Attachment with Cross-Lags | | | | | | |
| --- | --- | --- | --- | --- | --- | --- |
| **Model 1: Cross-Lags Between State Attachment Security and Avoidance** | | | | | | |
| Within-Person Effects | β_unstandardized_ [95% CrI] | | β_standardized_ [95% CrI] | | | |
| State Attachment Security 🡪 Security | **0.328 [0.267, 0.388]** | | **0.325 [0.286, 0.360]** | | | |
| State Attachment Security 🡪 Avoidance | **-0.142 [-0.214, -0.068]** | | **-0.083 [-0.112, -0.053]** | | | |
| State Attachment Avoidance 🡪 Avoidance | **0.275 [0.218, 0.330]** | | **0.276 [0.240, 0.308]** | | | |
| State Attachment Avoidance 🡪 Security | **-0.039 [-0.075, -0.003]** | | **-0.036 [-0.069, -0.003]** | | | |
| *R*^2^ State Attachment Security | .209 | | | | | |
| *R*^2^ State Attachment Avoidance | .174 | | | | | |
|  | State Attachment Security 🡪 Avoidance | | | State Attachment Avoidance 🡪 Security | | |
| Between-Person Predictors | β_unstandardized_ [95% CrI] | β [95% CrI] | β_unstandardized_ [95% CrI] | | β_standardized_ [95% CrI] | |
| Trait Attachment Avoidance | -0.032 [-0.107, 0.042] | -0.090 [-0.298, 0.115] | -0.026 [-0.062, 0.011] | | -0.120 [-0.273, 0.054] | |
| Trait Attachment Anxiety | -0.002 [-0.055, 0.051] | -0.009 [-0.202, 0.190] | -0.019 [-0.047, 0.009] | | -0.120 [-0.286, 0.054] | |
| % Time Spent Alone | 0.238 [-0.132, 0.613] | 0.130 [-0.072, 0.333] | 0.030 [-0.155, 0.216] | | 0.027 [-0.145, 0.191] | |
| Romantic Relationship Status | -0.032 [-0.160, 0.099] | -0.047 [-0.244, 0.139] | -0.023 [-0.095, 0.048] | | -0.057 [-0.234, 0.114] | |
| *R*^2^ | .059 | | .057 | | | |
| **Model 2: Cross-Lags Between State Attachment Security and Anxiety** | | | | | | |
| Within-Person Effects | β_unstandardized_ [95% CrI] | | β_standardized_ [95% CrI] | | | |
| State Attachment Security🡪 Security | **0.322 [0.263, 0.381]** | | **0.322 [0.286, 0.357]** | | | |
| State Attachment Security🡪 Anxiety | -0.094 [-0.186, 0.022] | | **-0.042 [-0.075, -0.006]** | | | |
| State Attachment Anxiety🡪 Anxiety | **0.298 [0.244, 0.350]** | | **0.295 [0.260, 0.331]** | | | |
| State Attachment Anxiety🡪 Security | 0.017 [-0.010, 0.045] | | 0.020 [-0.011, 0.049] | | | |
| *R*^2^ State Attachment Security | .189 | | | | | |
| *R*^2^ State Attachment Anxiety | .167 | | | | | |
|  | State Attachment Security 🡪 Anxiety | | | State Attachment Anxiety 🡪 Security | | |
| Between-Person Predictors | β_unstandardized_ [95% CrI] | β_standardized_ [95% CrI] | | β_unstandardized_ [95% CrI] | | β_standardized_ [95% CrI] |
| Trait Attachment Avoidance | 0.031 [-0.056, 0.118] | 0.079 [-0.135, 0.294] | | -0.001 [-0.029, 0.026] | | -0.010 [-0.290, 0.237] |
| Trait Attachment Anxiety | -0.018 [-0.088, 0.048] | -0.060 [-0.272, 0.166] | | 0.001 [-0.020, 0.023] | | 0.014 [-0.244, 0.291] |
| % Time Spent Alone | 0.086 [-0.397, 0.582] | 0.042 [-0.200, 0.260] | | 0.097 [-0.037, 0.242] | | 0.187 [-0.071, 0.460] |
| Romantic Relationship Status | 0.057 [-0.104, 0.228] | 0.076 [-0.142, 0.273] | | 0.036 [-0.016, 0.093] | | 0.188 [-0.091, 0.423] |
| *R*^2^ | .055 | | | .122 | | |
| **Model 3: Cross-Lags Between State Attachment Anxiety and Avoidance** | | | | | | |
| Within-Person Effects | β_unstandardized_ [95% CrI] | | β_standardized_ [95% CrI] | | | |
| State Attachment Anxiety🡪 Anxiety | **0.309 [0.254, 0.361]** | | **0.309 [0.273, 0.343]** | | | |
| State Attachment Anxiety 🡪 Avoidance | -0.037 [-0.084, 0.009] | | **-0.051 [-0.085, -0.017]** | | | |
| State Attachment Avoidance 🡪 Avoidance | **0.288 [0.232, 0.342]** | | **0.288 [0.252, 0.325]** | | | |
| State Attachment Avoidance 🡪 Anxiety | 0.037 [-0.024, 0.099] | | 0.030 [-0.005, 0.064] | | | |
| *R*^2^ State Attachment Anxiety | .173 | | | | | |
| *R*^2^ State Attachment Avoidance | .174 | | | | | |
|  | State Attachment Anxiety 🡪 Avoidance | | | State Attachment Avoidance 🡪 Anxiety | | |
| Between-Person Predictors | β_unstandardized_ [95% CrI] | β_standardized_ [95% CrI] | | β_unstandardized_ [95% CrI] | | β_standardized_ [95% CrI] |
| Trait Attachment Avoidance | -0.008 [-0.062, 0.046] | -0.034 [-0.246, 0.180] | | 0.028 [-0.037, 0.090] | | 0.095 [-0.123, 0.310] |
| Trait Attachment Anxiety | 0.012 [-0.029, 0.055] | 0.065 [-0.155, 0.275] | | 0.020 [-0.027, 0.067] | | 0.091 [-0.120, 0.287] |
| % Time Spent Alone | -0.047 [-0.336, 0.235] | -0.037 [-0.246, 0.188] | | -0.138 [-0.462, 0.189] | | -0.092 [-0.303, 0.123] |
| Romantic Relationship Status | -0.020 [-0.130, 0.084] | -0.042 [-0.251, 0.183] | | 0.023 [-0.096, 0.141] | | 0.041 [-0.166, 0.253] |
| *R*^2^ | .046 | | | .064 | | |
|  | | | | | | |

| **Table 1B**  Sensitivity Analysis I for Sample II: Unstandardized and Standardized Cross-Lags Between State Attachment Dimensions and Associations of Trait Attachment with Cross-Lags | | | | | | |
| --- | --- | --- | --- | --- | --- | --- |
| Model 1: Cross-Lags Between State Attachment Security and Avoidance | | | | | | |
| Within-Person Effects | β_unstandardized_ [95% CrI] | | β_standardized_ [95% CrI] | | | |
| State Attachment Security 🡪 Security | **0.310 [0.240, 0.377]** | | **0.316 [0.275, 0.349]** | | | |
| State Attachment Security 🡪 Avoidance | -0.099 [-0.211, 0.016] | | **-0.093 [-0.131, -0.056]** | | | |
| State Attachment Avoidance 🡪 Avoidance | **0.284 [0.221, 0.344]** | | **0.281 [0.244, 0.316]** | | | |
| State Attachment Avoidance 🡪 Security | -0.010 [-0.055, 0.036] | | -0.028 [-0.058, 0.003] | | | |
| *R*^2^ State Attachment Security | .232 | | | | | |
| *R*^2^ State Attachment Avoidance | .249 | | | | | |
|  | State Attachment Security 🡪 Avoidance | | | State Attachment Avoidance 🡪 Security | | |
| Between-Person Predictors | β_unstandardized_ [95% CrI] | β_standardized_ [95% CrI] | β_unstandardized_ [95% CrI] | | β_standardized_ [95% CrI] | |
| Trait Attachment Avoidance | -0.017 [-0.150, 0.112] | -0.029 [-0.233, 0.189] | -0.016 [-0.061, 0.028] | | -0.070 [-0.261, 0.120] | |
| Trait Attachment Anxiety | 0.030 [-0.058, 0.119] | 0.060 [-0.113, 0.224] | -0.011 [-0.045, 0.022] | | -0.058 [-0.220, 0.114] | |
| % Time Spent Alone | 0.259 [-0.423, 0.947] | 0.056 [-0.090, 0.199] | 0.001 [-0.279, 0.283] | | 0.001 [-0.155, 0.160] | |
| Romantic Relationship Status | 0.036 [-0.194, 0.268] | 0.029 [-0.157, 0.203] | -0.058 [-0.149, 0.032] | | -0.123 [-0.306, 0.065] | |
| *R*^2^ | .034 | | .044 | | | |
| **Model 2: Cross-Lags Between State Attachment Security and Anxiety** | | | | | | |
| Within-Person Effects | β_unstandardized_ [95% CrI] | | β_standardized_ [95% CrI] | | | |
| State Attachment Security🡪 Security | **0.323 [0.257, 0.386]** | | **0.319 [0.289, 0.347]** | | | |
| State Attachment Security🡪 Anxiety | **-0.142 [-0.229, -0.049]** | | **-0.067 [-0.096, -0.042]** | | | |
| State Attachment Anxiety🡪 Anxiety | **0.369 [0.316, 0.419]** | | **0.372 [0.341, 0.406]** | | | |
| State Attachment Anxiety🡪 Security | -0.014 [-0.036, 0.007] | | -0.007 [-0.032, 0.021] | | | |
| *R*^2^ State Attachment Security | .196 | | | | | |
| *R*^2^ State Attachment Anxiety | .208 | | | | | |
|  | State Attachment Security 🡪 Anxiety | | | State Attachment Anxiety 🡪 Security | | |
| Between-Person Predictors | β_unstandardized_ [95% CrI] | β_standardized_ [95% CrI] | | β_unstandardized_ [95% CrI] | | β_standardized_ [95% CrI] |
| Trait Attachment Avoidance | -0.015 [-0.098, 0.070] | -0.049 [-0.308, 0.218] | | 0.006 [-0.007, 0.020] | | 0.148 [-0.177, 0.408] |
| Trait Attachment Anxiety | -0.007 [-0.065, 0.052] | -0.027 [-0.237, 0.195] | | -0.003 [-0.014, 0.008] | | -0.100 [-0.388, 0.217] |
| % Time Spent Alone | 0.173 [-0.288, 0.637] | 0.073 [-0.116, 0.263] | | -0.026 [-0.131, 0.099] | | -0.079 [-0.431, 0.289] |
| Romantic Relationship Status | -0.084 [-0.246, 0.077] | -0.132 [-0.364, 0.118] | | -0.005 [-0.031, 0.021] | | -0.058 [-0.380, 0.225] |
| *R*^2^ | .066 | | | .127 | | |
| **Model 3: Cross-Lags Between State Attachment Anxiety and Avoidance** | | | | | | |
| Within-Person Effects | β_unstandardized_ [95% CrI] | | β_standardized_ [95% CrI] | | | |
| State Attachment Anxiety🡪 Anxiety | **0.378 [0.323, 0.431]** | | **0.377 [0.344, 0.410]** | | | |
| State Attachment Anxiety 🡪 Avoidance | 0.026 [-0.015, 0.067] | | **0.049 [0.015, 0.080]** | | | |
| State Attachment Avoidance 🡪 Avoidance | **0.300 [0.239, 0.359]** | | **0.304 [0.267, 0.338]** | | | |
| State Attachment Avoidance 🡪 Anxiety | 0.014 [-0.041, 0.069] | | 0.012 [-0.015, 0.040] | | | |
| *R*^2^ State Attachment Anxiety | .212 | | | | | |
| *R*^2^ State Attachment Avoidance | .225 | | | | | |
|  | State Attachment Anxiety 🡪 Avoidance | | | State Attachment Avoidance 🡪 Anxiety | | |
| Between-Person Predictors | β_unstandardized_ [95% CrI] | β_standardized_ [95% CrI] | | β_unstandardized_ [95% CrI] | | β_standardized_ [95% CrI] |
| Trait Attachment Avoidance | 0.029 [-0.018, 0.076] | 0.118 [-0.073, 0.294] | | -0.005 [-0.060, 0.046] | | -0.030 [-0.306, 0.276] |
| Trait Attachment Anxiety | **0.054 [0.021, 0.087]** | **0.256 [0.104, 0.387]** | | 0.004 [-0.037, 0.043] | | 0.024 [-0.223, 0.306] |
| % Time Spent Alone | 0.110 [-0.132, 0.346] | 0.057 [-0.065, 0.186] | | -0.079 [-0.402, 0.241] | | -0.058 [-0.324, 0.163] |
| Romantic Relationship Status | **0.125 [0.040, 0.212]** | **0.245 [0.078, 0.396]** | | -0.058 [-0.169, 0.048] | | -0.158 [-0.418, 0.139] |
| *R*^2^ | .160 | | | .089 | | |
|  | | | | | | |
