# Supplementary material for: Temporal Dynamics Between State Attachment Security, Avoidance, and Anxiety: Insights into Everyday Attachment System Functioning
Source: Pers Soc Psychol Bull. 2025 May 21;52(8):2382–98. doi: 10.1177/01461672251333472 (PMC13310265; doi:10.1177/01461672251333472)
Supplement: sj-docx-4-psp-10.1177_01461672251333472 – Supplemental material for Temporal Dynamics Between State Attachment Security, Avoidance, and Anxiety: Insights into Everyday Attachment System Functioning [file sj-docx-4-psp-10.1177_01461672251333472.docx]

**Supplemental Material 4: Preregistered Sensitivity Analysis II: All Three State Attachment Dimensions at Previous Moment Predict One State Attachment Dimension at Current Moment**

| **Table S4A**  Sensitivity Analysis II for Sample I: Unstandardized Cross-Lags Between State Attachment Dimension and Unstandardized Associations of Trait Attachment with Cross-Lags | | |
| --- | --- | --- |
| **Model 1: Cross-Lags Between State Attachment Security as Outcome** | | |
| Within-Person Effects | β_unstandardized_ [95% CrI] | β_standardized_ [95% CrI] |
| State Attachment Anxiety 🡪 Security | 0.015 [-0.016, 0.045] | 0.017 [-0.024, 0.053] |
| State Attachment Avoidance 🡪 Security | **-0.114 [-0.171, -0.057]** | **-0.142 [-0.186, -0.103]** |
| State Attachment Security 🡪 Security | **0.338 [0.265, 0.404]** | **0.345 [0.313, 0.379]** |
| *R*^2^ | .345 | |
| **Model 2: Cross-Lags Between State Attachment Avoidance** | | |
| Within-Person Effects | β_unstandardized_ [95% CrI] | β_standardized_ [95% CrI] |
| State Attachment Security 🡪 Avoidance | **-0.207 [-0.312, -0.103]** | **-0.156 [-0.194, -0.119]** |
| State Attachment Anxiety 🡪 Avoidance | **-0.105 [-0.168, -0.039]** | **-0.121 [-0.161, -0.077]** |
| State Attachment Avoidance 🡪 Avoidance | **0.230 [0.155, 0.303]** | **0.226 [0.169, 0.276]** |
| *R*^2^ | .307 | |
| **Model 3: Cross-Lags Between State Attachment Anxiety as Outcome** | | |
| Within-Person Effects | β_unstandardized_ [95% CrI] | β_standardized_ [95% CrI] |
| State Attachment Security 🡪 Anxiety | -0.064 [-0.194, 0.071] | -0.012 [-0.056, 0.038] |
| State Attachment Avoidance 🡪 Anxiety | -0.005 [-0.093, 0.086] | -0.027 [-0.068, 0.014] |
| State Attachment Anxiety 🡪 Anxiety | **0.290 [0.214, 0.361]** | **0.291 [0.245, 0.346]** |
| *R*^2^ | .293 | |
|  | | |

| **Table S4B**  Sensitivity Analysis II for Sample II: Standardized Cross-Lags Between State Attachment Dimension and Standardized Associations of Trait Attachment with Cross-Lags | | |
| --- | --- | --- |
| **Model 1: Cross-Lags Between State Attachment Security as Outcome** | | |
| Within-Person Effects | β_unstandardized_ [95% CrI] | β_standardized_ [95% CrI] |
| State Attachment Anxiety 🡪 Security | -0.017 [-0.039, 0.003] | -0.025 [-0.052, 0.009] |
| State Attachment Avoidance 🡪 Security | **-0.063 [-0.106, -0.020]** | **-0.083 [-0.114, -0.057]** |
| State Attachment Security 🡪 Security | **0.302 [0.226, 0.374]** | **0.306 [0.267, 0.356]** |
| *R*^2^ | .262 | |
| **Model 2: Cross-Lags Between State Attachment Avoidance** | | |
| Within-Person Effects | β_unstandardized_ [95% CrI] | β_standardized_ [95% CrI] |
| State Attachment Security 🡪 Avoidance | **-0.152 [-0.282, -0.016]** | **-0.128 [-0.158, -0.084]** |
| State Attachment Anxiety 🡪 Avoidance | 0.022 [-0.016, 0.059] | **0.038 [0.008, 0.068]** |
| State Attachment Avoidance 🡪 Avoidance | **0.268 [0.201, 0.333]** | **0.269 [0.230, 0.302]** |
| *R*^2^ | .301 | |
| **Model 3: Cross-Lags Between State Attachment Anxiety as Outcome** | | |
| Within-Person Effects | β_unstandardized_ [95% CrI] | β_standardized_ [95% CrI] |
| State Attachment Security 🡪 Anxiety | **-0.167 [-0.266, -0.073]** | **-0.070 [-0.097, -0.038]** |
| State Attachment Avoidance 🡪 Anxiety | -0.008 [-0.070, 0.052] | 0.005 [-0.021, 0.035] |
| State Attachment Anxiety 🡪 Anxiety | **0.402 [0.343, 0.455]** | **0.397 [0.358, 0.429]** |
| *R*^2^ | .272 | |
